# Pleura organoid transplantation facilitates visceral pleural repair through CD133^+^ progenitor cell-mediated regeneration

**DOI:** 10.1038/s12276-026-01825-w

**Published:** 2026-09-03

**Authors:** Biao Deng, Pengli Huang, Qu Wang, Ye He, Liuping Gong, Chunyuan Chen, Yanxia Wu, Zhenghao Lu, Zhu Liang

**Affiliations:** 1 Department of Thoracic, Affiliated Hospital of Guangdong Medical University, Zhanjiang, China; 2 GDMU-JingKe Biotech Joint Laboratory of Organoids, Affiliated Hospital of Guangdong Medical University, Zhanjiang, China; 3 Chengdu OrganoidMed Medical Laboratory, West China Health Valley, Chengdu, China; 4 Department of Pathology, Affiliated Hospital of Guangdong Medical University, Zhanjiang, China

**Keywords:** Stem-cell research, Respiratory tract diseases, Predictive markers, Mechanisms of disease

## Abstract

Persistent pulmonary air leakage following thoracic surgery remains a major clinical challenge. Here we establish murine and human pleura organoids as a novel regenerative therapy. Morphological and functional analyses confirm that these organoids retain mesothelial markers and secretory activity critical for pleural homeostasis. Xenotransplantation beneath the renal capsule of immunodeficient mice generates pleura-like structures with a mesothelial monolayer and collagen-elastin networks. In vitro wound healing and precision-cut lung slice models demonstrate enhanced mesothelial migration and organoid integration at injury sites. In vivo, transplantation into a murine pleural rupture model facilitates dynamic structural restoration, with continuous mesothelial layer reformation and sustained recovery of pleural barrier function, and attenuates acute inflammation and fibrosis. Transcriptomic and functional studies, notably human lung single-cell RNA sequencing analysis, identify CD133^+^ cells as a key subpopulation, exhibiting enhanced organoid-forming capacity and multilineage differentiation in vitro. Critically, CD133^+^ organoid transplantation significantly alleviates murine pleural rupture. This pioneers a biologically precise interventions, elucidating the important role of CD133^+^ cells in pleura repair, with transformative potential for thoracic regenerative medicine.

**Figure:**
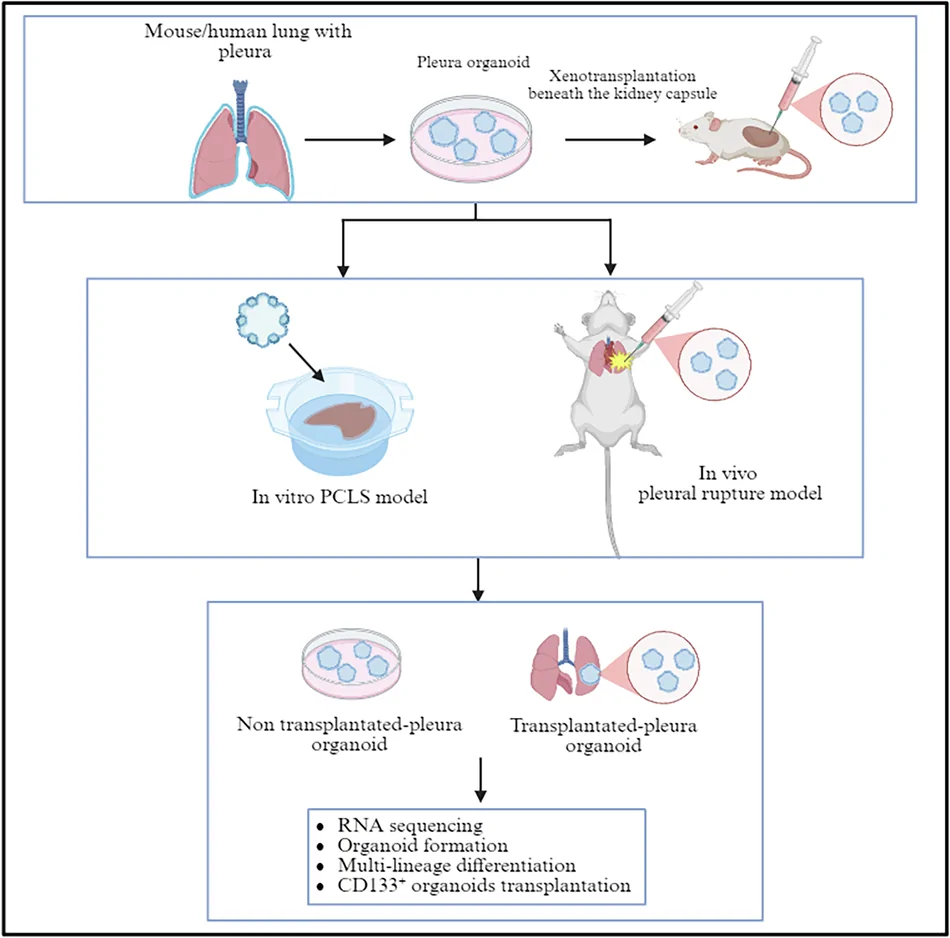

## Pleura organoids enhance healing in thoracic surgery recovery

Persistent pulmonary air leakage after thoracic surgery poses important clinical challenges, affecting many patients and leading to extended hospital stays and increased infection risks. This study introduces pleura organoids as a novel regenerative therapy for pulmonary air leakage, addressing the limitations of current treatments that focus on temporary sealing rather than true tissue regeneration. Researchers developed murine and human pleura organoid models enriched with CD133^+^ cells, hypothesizing their potential to engraft into damaged pleura and promote regeneration. Using precision-cut lung slices and murine models, they evaluated organoid integration and repair over time. Results showed that transplanted organoids formed pleura-like structures, enhancing mesothelial cell migration and reducing inflammation and fibrosis. CD133^+^ cells emerged as a key subpopulation, exhibiting stem cell-like properties and contributing to mesothelial regeneration. This summary was initially drafted using artificial intelligence, then revised and fact-checked by the author.

## Introduction

Persistent pulmonary air leakage (PAL) following thoracic surgery remains a critical clinical challenge, affecting 10–26% of patients and resulting in prolonged hospitalization, increased risk of infections and substantial healthcare burdens^1,2^. PAL occurs owing to incomplete healing of the visceral pleura, a thin, mesothelial-lined membrane that envelops the lungs. When the alveolar-pleural fistulae fail to seal, pathological air exchange occurs between the lung parenchyma and the pleural cavity. Current repair strategies, including synthetic polymer patches, biological grafts (for example, bovine pericardium) and chemical pleurodesis (for example, talc or bleomycin), primarily focus on physical sealing or inducing inflammatory adhesion^3–6^. Although these methods provide temporary relief, they often exacerbate tissue damage through foreign body reactions, chronic inflammation and fibrosis, ultimately leading to recurrent air leaks or pleural adhesions^7,8^. These outcomes highlight the urgent need for therapies that not only seal defects but also restore the native cellular and extracellular architecture of the pleura to enable functional regeneration.

Recent advances in regenerative medicine underscore the potential of cell-based therapies as promising alternatives. Stem cell transplantation, particularly involving mesenchymal stromal cells, has demonstrated efficacy in repairing bone, cartilage and neural tissues by promoting paracrine signaling and the differentiation of progenitor cells^9–12^. However, mesenchymal stromal cell-based approaches for pleural repair remain underexplored, limited and constrained by in site-specific engraftment and lineage commitment. Emerging organoid technology, which utilizes self-organizing stem cells to generate three-dimensional tissue mimics, offers a paradigm shift. Organoids replicate the structural and functional complexity of native tissues, facilitating the precise modeling of development, disease and repair^13^. For example, pancreatic islet organoids derived from induced pluripotent stem cells have successfully restored glucose homeostasis in diabetic models^14^, whereas intestinal organoids enhanced epithelial regeneration in ischemia–reperfusion injury by modulating local immune responses^15^. Similarly, liver organoids have mitigated fibrosis by repolarizing macrophages toward a proregenerative phenotype^16^. Despite these advancements, no studies have yet utilized organoid technology to address pleural injury, underscoring a notable gap in research. This is particularly important given the unique mesothelial–stromal interface of the pleura and its vulnerability to mechanical stress during respiratory cycles.

The visceral pleura consists of multiple layers: a superficial mesothelial monolayer, submesothelial connective tissue and elastic fiber networks that collectively maintain pulmonary integrity^17^. Repairing this stratified structure requires not only mesothelial regeneration but also the re-establishment of a functional tissue architecture, which current therapies inadequately address. Organoid transplantation, by providing tissue-specific progenitor cells and a supportive microenvironment, may offer a more comprehensive solution. Recent studies on lung organoids have demonstrated their capacity to model alveolar and airway repair^18,19^; nevertheless, pleural-specific organoids remain uncharacterized. A key barrier to progress is the lack of protocols for isolating and expanding pleural mesothelial progenitors while preserving their differentiation potential. Furthermore, the identity of regenerative cell subpopulations within the pleura and their mechanisms of action remain unknown. CD133 (prominin-1), a marker of stem and progenitor cells in various tissues^20–22^, has not yet been investigated in mesothelial contexts.

In this study, we establish the first murine and human pleura organoid models to investigate their reparative potential in visceral pleural injury. We hypothesize that pleura organoids, enriched with CD133^+^ cells, can engraft into damaged regions, contribute to functional mesothelium regeneration and orchestrate tissue architecture remodeling. Utilizing precision-cut lung slices (PCLS) and murine injury models, we evaluate organoid integration, dynamic structural repair and functional barrier restoration over time. Transcriptomic and functional analyses further elucidate the role of CD133^+^ cells in promoting this regeneration. This study pioneers a novel biological strategy for PAL treatment, unveiling mechanistic insights into mesothelial regeneration and bridging a critical gap between regenerative medicine and thoracic surgery. By addressing both cellular and structural deficits, pleura organoid transplantation emerges as a transformative approach to restore pulmonary homeostasis and mitigate postoperative complications.

## Materials and methods

### Reagents and chemicals

DMEM/F12 (cat. no. 11320082), Medium 199 (cat. no. 12340030), fetal bovine serum (FBS, cat. no. A5256701), penicillin–streptomycin solution (cat. no. 15140122), trypsin (cat. no. 25200072), DNase I solution (cat. no. 90083) and TrypLE (cat. no. 12563029) were purchased from Thermo Fisher Scientific. Growth factor reduced matrigel (cat. no. 356231) was purchased from Corning Incorporated (Corning). Phosphate-buffered saline (PBS; cat. no. C0221A), Triton X-100 (cat. no. P0096) and DAPI (cat. no. C1002) were purchased from Beyotime Biotechnology. Type I collagenase (cat. no. LS004196) and type IV collagenase (cat. no. LS004188) were purchased from Worthington. Recombinant RSPO1 (cat. no. 120-38), epidermal growth factor (EGF; cat. no. AF-100-15), fibroblast growth factor 10 (FGF-10; cat. no. 100-26) and Noggin (cat. no. 120-10C) were purchased from PeproTech.

### Experimental model details

For mouse organoid generation, 8–10-week-old C57BL/6 mice of both sexes were used. For injury and transplantation assays, 8–10-week-old immunodeficient NSG mice of both sexes were used. These strains were purchased from the Jackson Laboratory. Mice were maintained in virus-free conditions and were housed in groups. Animal experiments were conducted in accordance with international ethical guidelines and the National Institutes of Health Guide concerning the Care and Use of Laboratory Animals, and the experiments were carried out with the approval of the Animal Experimentation Ethics Committee of Guangdong Medical University. For human organoid generation, healthy lung tissue from patients undergoing lung resection were used. All experiments with human tissue were approved by the clinical research ethical committee of the Guangdong Medical University and we complied with all relevant ethical regulations.

### Method details

#### Mouse pleura organoids culture

C57BL/6 mouse was euthanized and intact lungs with trachea were isolated and suspended in collagenase solution at 1 mg/ml. After digestion for 30–60 min, the cells isolated from pleura were seeded in Matrigel (Corning) and cultured in pleura organoids medium (Medium 199 supplemented with 1% penicillin–streptomycin, GlutaMAX (2 mM, Peprotech), *N*-acetylcysteine (1 mM, Sigma), 2% B27 supplement (Gibco), 2% N2 supplement (Gibco), RSPO1 (100 ng/ml, Peprotech), EGF (10 ng/ml, Peprotech), FGF-10 (20 ng/ml, Preprotech), Noggin (100 ng/ml, Peprotech), 10% Wnt-3A conditioned medium, Rho-kinase inhibitor Y-27632 (10 µM, MCE), TGF-β type I receptor inhibitor A83-01 (2 μM, MCE), ascorbic acid (50 μg/ml, MCE), 1% ITS (Gibco), dexamethasone (200 nM, MCE) and retinoic acid (50 nM, MCE)). The medium was changed every 2–3 days, and subculture was conducted every week with a subculture ratio of 1:4 to 1:6.

#### Human pleura organoids culture

The surgically obtained healthy lung tissue with a surface covering visceral pleura was washed five to six times with Dulbecco’s PBS and carefully removed fat, fascia and blood clots. The tissue was then suspended in a 50-ml centrifuge tube by tying it with sterilized ophthalmic sutures, and the areas with surgical trauma and without pleural coverage were placed upward. The pleura-covered areas were just submerged in collagenase solution and digested for 30–60 min, then the cells isolated from pleura were seeded in Matrigel and cultured in pleura organoids medium (Medium 199 supplemented with 1% penicillin–streptomycin, GlutaMAX (2 mM), *N*-acetylcysteine (1 mM), nicotinamide (1 mM, Sigma), 2% B27 supplement, 2% N2 supplement, RSPO1 (100 ng/ml), EGF (10 ng/ml), FGF-10 (20 ng/ml), Noggin (100 ng/ml), 10% Wnt-3A conditioned medium, Y-27632 (10 µM), A83-01 (2 μM), ascorbic acid (50 μg/ml), prostaglandin E2 (PGE2; 2 μM, MCE), 1% ITS, dexamethasone (200 nM) and retinoic acid (50 nM)). The medium was changed every 2–3 days, and subculture was conducted biweekly with a subculture ratio of 1:2 to 1:4.

#### Lung organoids culture

After obtaining cells of surface pleural origin, the remaining lung tissues were cut into small pieces of about 1 mm^3^ and transferred into a collagenase solution. After digestion for 20–30 min, the fragments from the airway and alveolar were collected and embedded in Matrigel and cultured in lung organoids medium (advanced DMEM/F12 supplemented with GlutaMAX (2 mM), *N*-acetylcysteine (1 mM), nicotinamide (1 mM), 1% penicillin–streptomycin, 2% B27 supplement, 2% N2 supplement, RSPO1 (100 ng/ml), EGF (50 ng/ml), fibroblast growth factor 7 (FGF-7; 25 ng/ml, Preprotech), FGF-10 (100 ng/ml), 10% Wnt-3A conditioned medium, 10 µM Y-27632, 50 nM dexamethasone, 100 nM 8-bromo-cAMP (MCE) and 100 μM IBMX (MCE)). Detailed recipes for media composition are described in Supplementary Table 1. Media were changed every 2–3 days, with murine and human organoid subcultures being conducted every week and biweekly, respectively. The subculture ratio was 1:4 to 1:6 for murine organoids, whereas it was 1:2 to 1:4 for human organoids.

#### Wound-scratch assay

Human pleura organoids were disgested into single cells and seeded on the lower chamber of a 24-well Transwell plate at a concentration of 1 × 10^5^ ml^−1^ and were grown to confluence. Then, the scratch wound was made with a 200-μl pipette tip, TNF-α (50 ng/ml) was added to the lower chamber to simulate the inflammatory environment when the wound occurs and pleura organoid grafts or an equivalent volume of medium was added into the upper compartment of the insert. After 24 h of incubation, the wounds were observed under a microscope. The diameter of the initial wound was also measured, and the relative wound closure was determined on the basis of the initial wound diameter.

#### Mouse PCLS

The NSG mouse was killed, and intact lungs with trachea were isolated. Next, 1 ml warm (40 °C) 2% low-melting-point agarose was loaded into a 1-ml syringe and slowly injected into the lungs through the trachea, and the trachea was tied to prevent agarose leakage. After the agarose fully solidified, lungs were glued onto a specimen holder, and slices were made with a VT 1200S vibratome (Leica). The parameters of the slicer are set as follows: tissue thickness, 200 µm; amplitude, 1.2 mm; frequency, 100 Hz; and feed rate, 0.12 mm/s. Lung slices were placed into the upper compartment of a 24-well Transwell plate and cultured in the air–liquid method.

#### In vitro organoids transplantation based on the PCLS model

After successfully culturing PCLS, a surgical blade was used to create a notch in the pleural region on the surface of PCLS under a dissecting microscope to simulate pleural rupture. Next, human pleura organoids in good growth condition were taken, digested into single cells, labeled with PKH26 fluorescent dye at a concentration of 10 μM, counted and then aspirated. In total, 2 × 10^4^ or 5 × 10^4^ cells were resuspended with 10 μl of medium containing 10% Matrigel and added dropwise to the broken area of the PCLS to simulate organoid transplantation. The PCLS were rested for 30 min at 37 °C, and then 90 μl of medium was added to each well along the wall of the wells and incubated at 37 °C. The fluid was changed for 2–3 days. At 7 days after transplantation, the PCLS was washed with Dulbecco’s PBS to remove unintegrated organoid cells, and the red fluorescence of PKH26 was detected to determine whether the pleura organoids were able to integrate into the area of pleural breakage after transplantation.

#### Histology and immunofluorescence

Tissues or organoids were fixed in 4% paraformaldehyde, dehydrated and embedded in paraffin. Sections were subjected to hematoxylin and eosin (H&E) or immunofluorescence staining. Immunofluorescence staining was performed according to standard protocols on 5-µm sections. The sections were incubated in blocking buffer (0.25% Triton X-100 and 5% normal goat serum) for 1 h at room temperature (RT), then washed three times in PBS. The organoids were incubated with primary antibodies in antibody dilution buffer (0.25% Triton X-100 and 1% BSA in PBS) overnight at 4 °C. The sections were then washed with PBS five times for 10 min each. Rabbit anti-Ki67 (1:200, cat. no. 27309-1-AP, Proteintech), rabbit anti-epithelial cell adhesion molecule (EPCAM; 1:200, cat. no. 21050-1-AP, Proteintech), rabbit anti-MSLN (1:200, cat. no. A14099, ABclonal Technology), rabbit anti-calretinin (CR; 1:200, cat. no. 12278-1-AP, Proteintech), rabbit anti-vimentin (VIM; 1:200, cat. no. 10366-1-AP, Proteintech), mouse anti-CD133 (1:200, cat. no. 66666-1-Ig, Proteintech) and mouse anti-HNA (1:100, cat. no. ab191181, Abcam) were used as primary antibodies. Then the sections were incubated with secondary antibodies in antibody dilution buffer for 1 h at RT, then washed with PBS five times for 10 min each. CoraLite488-conjugated Goat Anti-Rabbit IgG (1:500, cat. no. SA00013-2, Proteintech), Cy3-conjugated Goat Anti-Rabbit IgG (1:500, cat. no. SA00009-2, Proteintech) and Cy3-conjugated Goat Anti-Mouse IgG (1:500, cat. no. SA00009-1, Proteintech) were used as secondary antibodies. Nuclei were stained with DAPI for 30 min at RT. Immunofluorescence images were acquired using a confocal microscope (Nikon).

#### Heterotopic transplantation of human pleura organoids

Human pleura organoids were collected and digested into single cells, then 5 × 10^5^ cells were resuspended with 50 μl pleura organoids medium containing 50% Matrigel and 10 µM Y-27632 and carefully transferred into an Eppendorf tube. The collected cells were transplanted into the renal subcapsular space of 8-week-old immunodeficient male NSG mice. In brief, the mice were anesthetized by intraperitoneal injection of 50 mg/kg sodium pentobarbital, after which the kidney was exposed via a dorsal flank incision. After incision of the host kidney capsule (approximately 2 mm) with a 23-gauge syringe needle, a PE50 tube containing 5 × 10^5^ cells was carefully placed under the kidney capsule. The cells were delivered by carefully blowing through the other side of the PE50 tube. Mice were killed 21 days after transplantation.

#### Orthotopic transplantation of human pleura organoids

Human pleura organoids were collected and digested into single cells, then 5 × 10^5^ cells were resuspended with 50 μl pleura organoids medium containing 50% Matrigel and 10 µM Y-27632 and carefully transferred into an Eppendorf tube. In brief, the mice were anesthetized with sodium pentobarbital. Then, the cell suspension was aspirated with a 1-ml insulin syringe, and the needle was inserted in the right fifth/sixth intercostal space of the mouse, penetrating the chest wall and then continuing to puncture the visceral pleura on the lung surface to simulate the rupture of the visceral pleura caused by surgery. After that, the syringe was slightly returned, and cells were delivered into the pleural cavity. Mice in the model group were transplanted with an equal volume of the same vector without organoid cells after modeling. Mice were killed 21 days after transplantation.

### RNA-seq

Transplanted pleura organoids (*n* = 3) and nontransplanted pleura organoids (*n* = 3) were collected, and TRIzol reagent (cat. no. 10296028, Thermo Fisher Scientific) was used to extract the total RNA from the organoids. RNA sequencing (RNA-seq) was performed by OE Biotech. In brief, all RNA samples underwent quality control analysis using Fast QC (version 0.10.1). Sequencing libraries were prepared using a TruSeq Stranded Total RNA Library Prep Kit (15032612, Illumina). CBot (HiSeq2500) was used to generate clusters and the HiSeq2500 sequencing platform (Illumina) was used to generate pairend 150-bp reads. Valid clean data were obtained using Trimadap to filter out unqualified sequences, whereas HISAT2 was used for reference genome alignment. On the basis of HISAT2 alignment results, transcripts were reconstructed using StringTie (John Hopkins University), and expression levels of all genes in each sample were calculated. The computation of differential gene expression was completed by the negative binomial model implemented in the Bioconductor package, edgeR. A fold change (FC) ≥2 or FC ≤−2 with a *q* value <0.05 was considered significant.

### scRNA-seq data processing and analysis

Single-cell RNA-seq (scRNA-seq) data of human lung tissue were obtained from the Gene Expression Omnibus (GEO) database. A total of 116,852 cells was included in the analysis. The raw count matrices were processed using the Seurat package (v5.0.3) in R. Quality control screening criteria included: (1) removing cells with fewer than 300 detected genes, (2) excluding genes expressed in fewer than five cells and (3) discarding cells with a mitochondrial transcript proportion >20%, ribosomal content >3% or erythrocyte content <1%. High-quality cells were normalized using the LogNormalize method, and the transcript counts for each cell were scaled to 10,000. The top 2,000 highly variable genes were selected for principal component analysis, and the top 30 principal components were chosen for subsequent analysis. The Harmony algorithm was used to correct for potential batch effects between samples, and dimension reduction was performed using unified manifold approximation and projection (UMAP) and *t*-distributed stochastic neighborhood embedding (*t*-SNE). Unsupervised graph clustering was performed using the FindNeighbors and FindClusters functions in the Seurat software, with the resolution parameter set to 0.8. Subsequently, cell types were annotated on the basis of the expression profiles of classic marker genes and manual verification of expression patterns. The FindAllMarkers function (Wilcoxon rank-sum test, |log₂FC| ≥0.25, corrected *P* < 0.05) was used to identify cell-cluster-specific marker genes, and the top-ranking marker genes were visualized using heat maps and scatter plots. Differentiation trajectories were inferred for stem cell subpopulations using Monocle3 software (v1.3.4). The dimensionality reduction was performed using UMAP and trajectory plots constructed using the ‘learn_graph()‘ function. The relative simulated time values were assigned with the cell cluster exhibiting the highest expression of stem cell marker genes as the root node.

### Statistical analysis

Analyses were performed via GraphPad Prism 8.0 software (GraphPad Software), with significance set to *P* < 0.05. The number of samples and independent biological experimental repeats are indicated in the figure legends. All the quantitative data are expressed as the means ± s.d. A two-tailed unpaired *t*-test was used to determine statistical significance between two groups, and one-way analysis of variance (ANOVA) was used to determine significant differences among multiple groups.

## Results

### Establishment of murine adult pleura organoid cultures

**Fig. 1:**
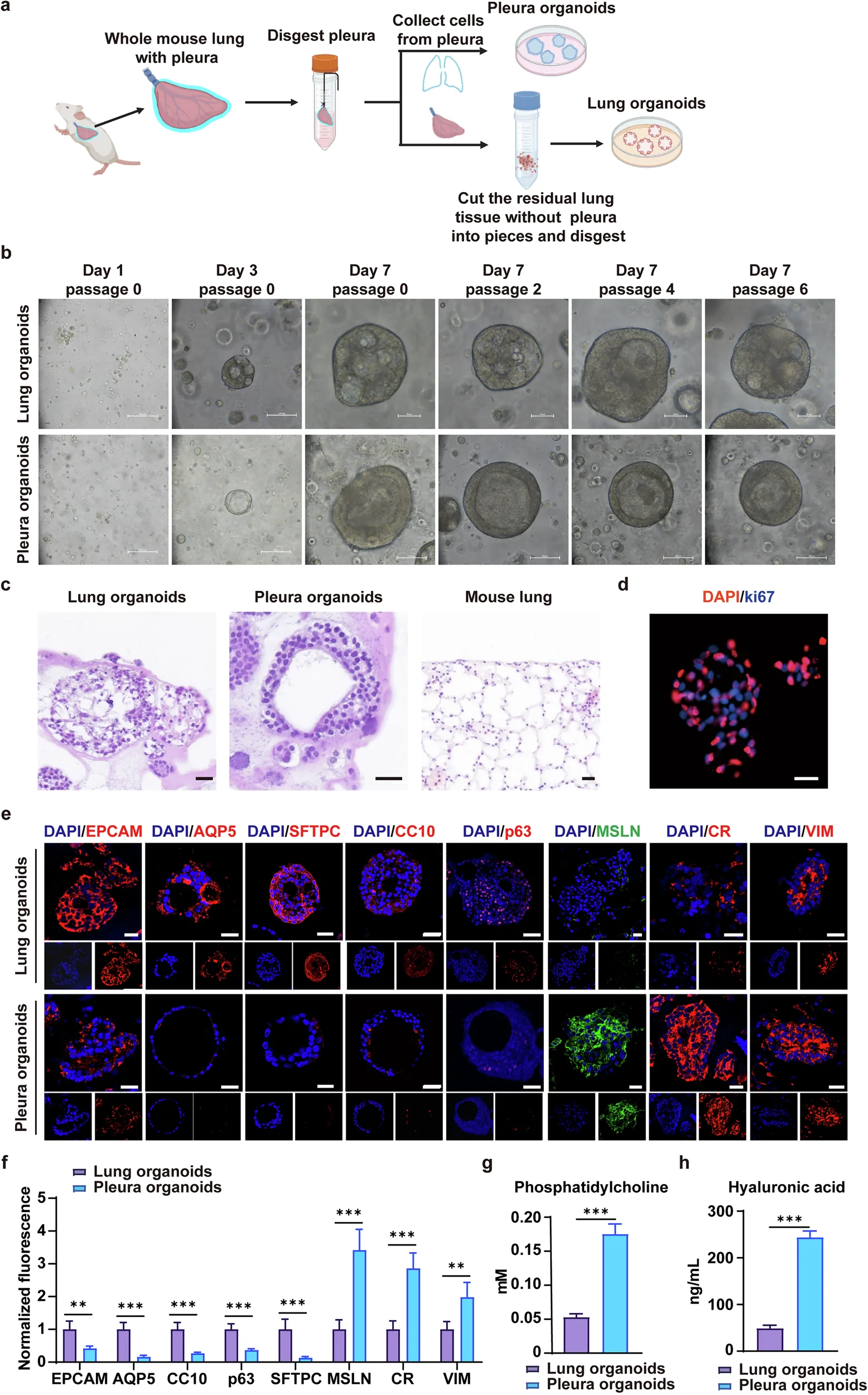
Establishment of murine pleura organoid cultures. **a** A schematic diagram of the workflows to establish murine pleura organoids and lung organoids. **b** Representative brightfield microscopy images of murine pleura organoids and lung organoids. Scale bar, 50 μm. **c** Representative H&E images of murine lung tissue, pleura organoids and lung organoids. Scale bar, 20 μm. **d** A representative image of IF staining of pleura organoids. Blue, DAPI; red, Ki67. Scale bars, 20 μm. **e** Representative images of IF staining of pleura organoids and lung organoids. Blue, DAPI; red, EPCAM; red, AQP5; red, SFTPC; red, CC10; red, p63; green, MSLN; red, CR; red, VIM. Scale bar, 20 μm. **f** The quantification of the normalized fluorescence intensity of the markers as described in **e**. *n* = 3 per group. **g**, **h** A comparison of phosphatidylcholine (**g**) and hyaluronic acid (**h**) contents in the medium of pleura organoids and lung organoids. *n* = 3 per group. The data are presented as the means ± s.d. Two-tailed unpaired *t*-tests were performed to assess the statistical significance of differences between two groups. **P < 0.01 and ***P < 0.001 versus the indicated group.

In the present study, organoids developed from lung tissues primarily focus on cell types, structures and functions related to alveolar and airway tissues^18,23^, whereas little attention has been given to the visceral pleura. We first attempted to construct pleura organoids using mouse lungs. To avoid contamination with parenchymal cell components, we isolated intact lung tissue without cutting, tied off the trachea and suspended the tissue in a digestive solution for optimal enzymatic digestion, carefully controlling the digestion time between 30 and 60 min. This approach ensured adequate recovery of pleural cells while preventing overdigestion, which could lead to dissociation of deeper parenchymal cells. Subsequently, we constructed pleura organoids using the isolated cells (Fig. 1a). After isolating cells of surface pleural origin, the remaining tissue from the excised pleural layer was minced into small pieces and digested to extract lung parenchymal cell components for constructing corresponding lung organoids. Key growth factors in the lung organoid medium included R-spondin, EGF, FGF-10, FGF-7 and Noggin, whereas the addition of dexamethasone and 8-bromo-cyclic AMP (an activator of protein kinase A) promoted alveolar differentiation and maturation^18,23^. After 1 week of incubation, cells from the lung parenchyma grew into honeycomb-like cystic clusters composed of cuboidal and thin cells, a morphology reminiscent of alveoli (Fig. 1b, c). Immunofluorescence staining results indicated that the epithelial cell marker EPCAM^19^ was widely expressed in lung organoids and canonical markers for AT1 cells (aquaporin-5, AQP5), AT2 cells (surfactant protein C, SFTPC)^24^, club cells (club cells 10 kDa secretory protein, CC10) and basal cells (tumor protein 63, p63)^23^ were also detected (Fig. 1e).

After screening a range of growth factors for their ability to induce pleural differentiation, we established a pleural differentiation protocol. In brief, cells collected from the pleura following enzymatic digestion were embedded in matrix gel to support three-dimensional culture. These cells were then cultured in pleura organoid medium containing pleural maturation additives (A83-01, ascorbic acid, retinoic acid and increased-dose dexamethasone) while removing or reducing the lung epithelial cell-supporting factors (EGF, FGF-10 and FGF-7) (Supplementary Table 1), thereby promoting the growth of mesothelial cells, the predominant cell type in the pleura. After 1 week of culture, pleural cells formed organoids exhibiting typical morphology characterized by thick walls and a central lumen, distinguishable from the alveolar-like morphology of lung organoids (Fig. 1b, c). Immunofluorescence analysis of proliferation marker protein Ki67 (Ki67) indicated that a high proportion of the pleura organoids retained proliferative activity, as evidenced by Ki67 positivity (Fig. 1d). Furthermore, the pleura organoids could be expanded over an extended period, exceeding 20 consecutive passages.

Compared with lung organoids, pleura organoids exhibited a notable decrease in the expression of epithelial cell marker (EPCAM), alveolar epithelium markers (AQP5 and SFTPC) and airway epithelium markers (CC10 and p63). By contrast, mesothelial cell markers mesothelin (MSLN) and CR^25^, as well as VIM, a marker found in mesenchymal cells and fibroblasts^26,27^, were significantly upregulated (Fig. 1e, f). Furthermore, ELISA results confirmed that pleura organoids secreted higher levels of phosphatidylcholine (Fig. 1g) and hyaluronic acid (Fig. 1h), substances typically secreted by mesothelial cells that primarily function to lubricate the pleural cavity. These findings indicate that pleura organoids generated through differential culture differ from matched lung organoids in both morphological structure and cellular composition, while largely preserving the morphological and functional characteristics of their original tissue.

### Establishment of human adult pleura organoid cultures

**Fig. 2:**
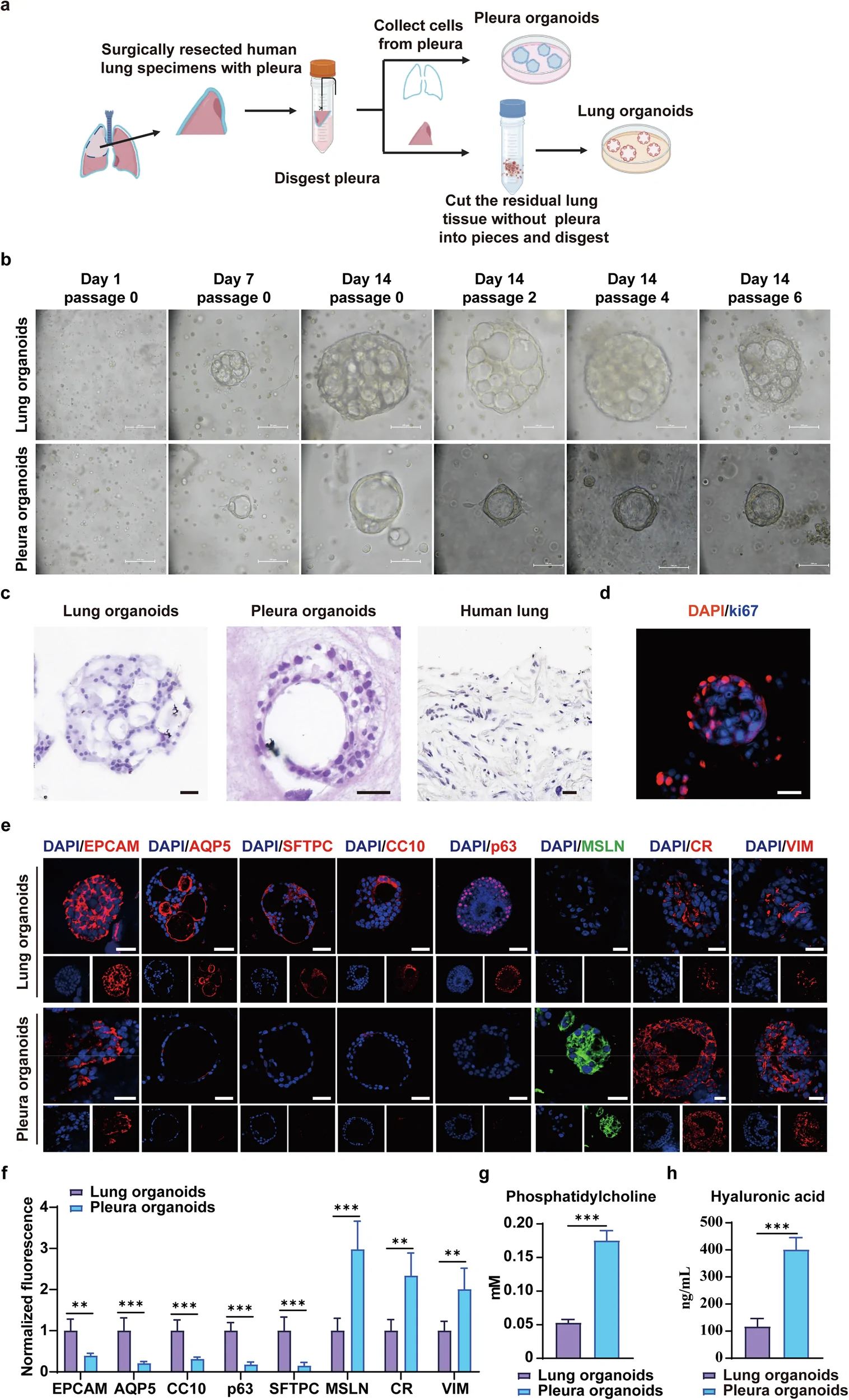
Establishment of human pleura organoid cultures. **a** A schematic diagram of the workflows to establish human pleura organoids and lung organoids. **b** The representative brightfield microscopy images of human pleura organoids and lung organoids. Scale bar, 100 μm. **c** Representative H&E images of human lung tissue, pleura organoids and lung organoids. Scale bar, 20 μm. **d** A representative image of IF staining of pleura organoids. Blue, DAPI; red, Ki67. Scale bars, 20 μm. **e** Representative images of IF staining of pleura organoids and lung organoids. Blue, DAPI; red, EPCAM; red, AQP5; red, SFTPC; red, CC10; red, p63; green, MSLN; red, CR; red, VIM. Scale bar, 20 μm. **f** The quantification of the normalized fluorescence intensity of the markers as described in **e**. *n* = 3 per group. **g**, **h** A comparison of phosphatidylcholine (**g**) and hyaluronic acid (**h**) contents in the medium of pleura organoids and lung organoids. *n* = 3 per group. The data are presented as the means ± s.d. Two-tailed unpaired *t*-tests were performed to assess the statistical significance of differences between two groups. **P < 0.01 and ***P < 0.001 versus the indicated group.

We next investigated whether our culture protocol could be adapted to human pleura organoids. We established human pleura organoids using surgically obtained healthy lung tissue that included a surface layer of visceral pleura, using a method similar to that used for constructing mouse pleura organoids (Fig. 2a). To establish human pleura organoids, we modified the murine pleura organoid medium by adding nicotinamide, PGE2 and GSK3 inhibitor CHIR99021, which are known to be essential for maintaining human organoids derived from other tissues^28–31^. Using this approach (Supplementary Table 1), human pleura organoids could be expanded over an extended period. After a 2-week incubation, human pleura organoids displayed a typical morphology characterized by thick walls and a central lumen, resembling murine pleura organoids (Fig. 2b, c). Immunofluorescence staining for Ki67 indicated that human pleura organoids maintained proliferative activity (Fig. 2d).

In addition, we characterized both lung and pleura organoids using immunofluorescence staining. Similar to murine organoids, human pleura organoids expressed a broader range of mesothelial cell markers, including MSLN and CR, as well as the fibroblast marker VIM. By contrast, lung organoids exhibited higher fluorescence intensity for the epithelial cell marker EPCAM, the alveolar epithelium markers AQP5 and SFTPC and the airway epithelium markers CC10 and p63 (Fig. 2e, f). ELISA results demonstrated that pleura organoids secreted higher levels of phosphatidylcholine (Fig. 2g) and hyaluronic acid (Fig. 2h) compared with lung organoids. These data suggest that human pleura organoids differ from lung organoids in histomorphology, specific marker expression and secretory function.

### Human pleura organoids form pleura-like structures after xenotransplantation

**Fig. 3:**
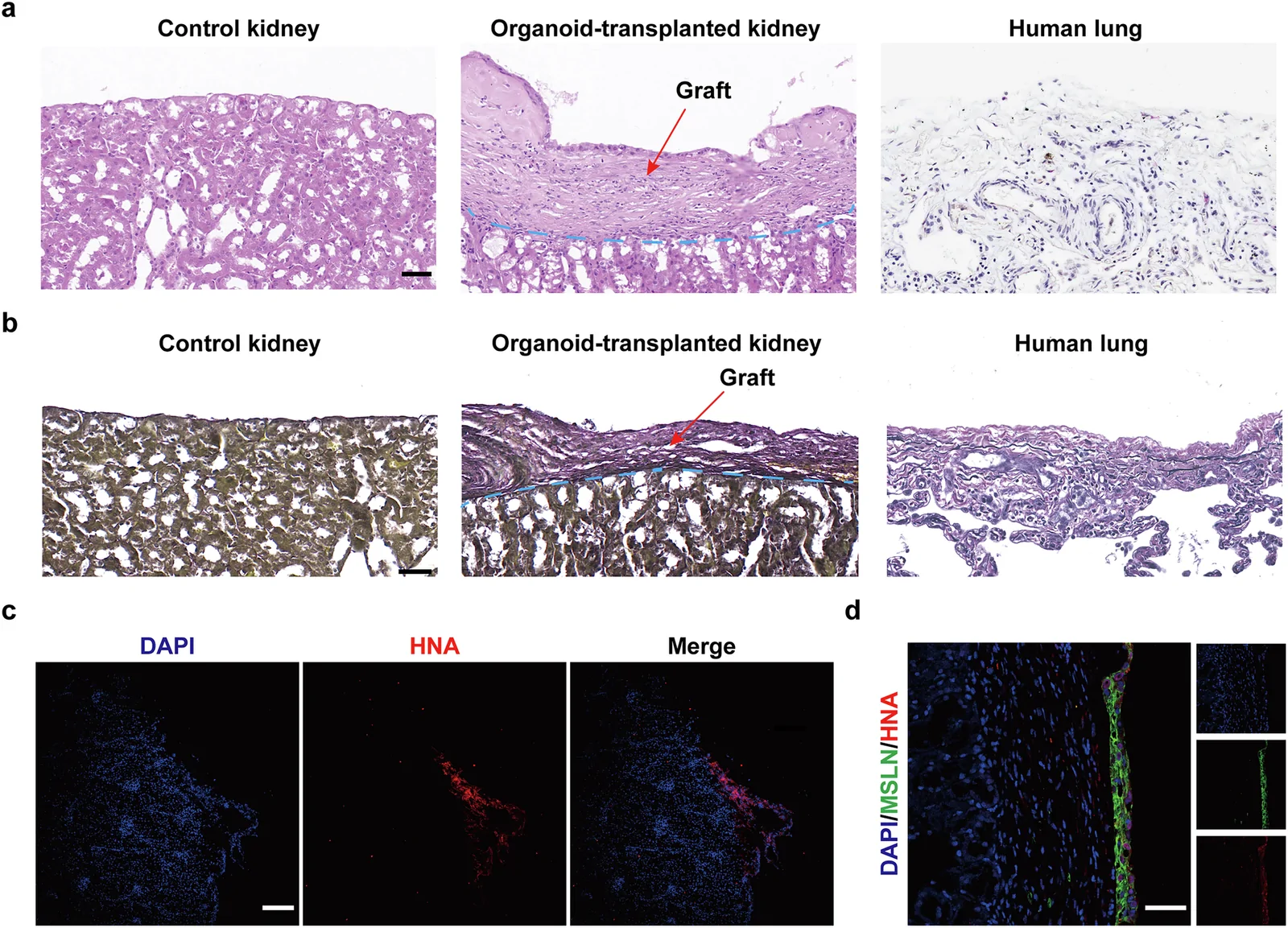
Human pleura organoids form pleura-like structures after xenotransplantation. **a** Representative H&E images of control kidney, organoid-transplanted kidney and parental human lung. Scale bar, 50 μm. **b** Representative images of EVG-Verhöeff staining of the control kidney, organoid-transplanted kidney and parental human lung. Scale bar, 50 μm. **c** Representative images of IF staining of organoid-transplanted kidney. Blue, DAPI; red, HNA. Scale bar, 200 μm. **d** Representative images of IF staining of organoid-transplanted kidney. Blue, DAPI; green, MSLN; red, HNA. Scale bar, 50 μm.

The transplantation of organoids into mouse tissues can enhance the maturation of organoid structure and function. In this study, we transplanted generated human pleura organoids beneath the kidney capsule of immunodeficient NSG mice to determine whether they could develop pleura-like structures under conditions of abundant blood supply and adequate nutrition. At 3 weeks post transplantation, a well-organized multilayered structure was observed at the site of the human pleura organoid transplantation, exhibiting similarities to the superficial pleural region of human lung tissue (Fig. 3a). Using EVG-Verhöeff staining, we demonstrated the distribution of collagen and elastic fibers within these multilayered structures, both of which are key components of the visceral pleura (Fig. 3b). To identify the cellular origin of the multilayered structures, we performed immunofluorescence staining for the human-derived cell marker human nuclear antigen (HNA). The results showed that cells in the region of the multilayered structures expressed HNA, confirming that these structures were formed by transplanted human pleura organoids rather than by proliferation of mouse tissue (Fig. 3c). As expected, we observed a continuous layer of mesothelial cells in the outermost layer of the multilayered structure formed by human pleura organoids (Fig. 3d), further validating the similarity of the multilayered structure to the visceral pleura. These findings suggest that xenotransplantation of human pleura organoids into the kidney capsule can generate a multilayered structure akin to the visceral pleura, containing essential cellular components (mesothelial cells) as well as mesenchymal constituents (collagen and elastic fibers). This provides a foundational basis for a strategy aimed at repairing pleural injuries through organoid transplantation.

### In vitro wound healing evaluation of pleura organoids transplantation

**Fig. 4:**
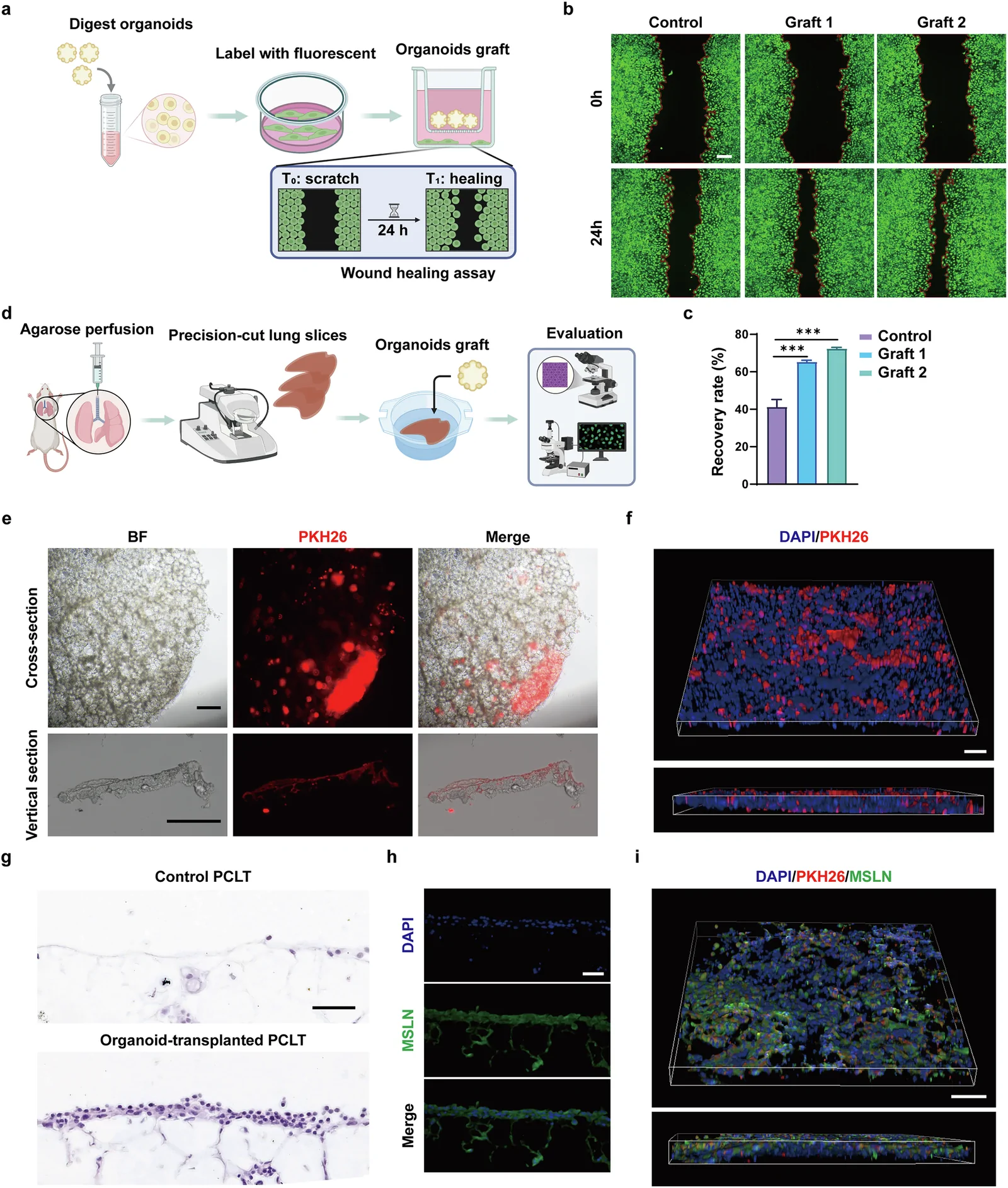
In vitro wound healing evaluation of pleura organoid transplantation. **a** A schematic diagram of the workflow of in vitro wound healing assay based on a Transwell model. **b** Representative fluorescence images of a wound healing assay in mesothelial cells of the control group and two organoid-transplanted groups. Mesothelial cells labeled by Calcein AM are presented in green. Scale bar, 200 μm. **c** The quantification of the wound healing assay as described in **b**. *n* = 3 per group. **d** A schematic diagram of the workflow to evaluate the effect of organoid transplantation in vitro based on a PCLS model. **e** A brightfield and fluorescence image of the cross-section (top row) and vertical section (bottom row) of the organoid-transplanted PCLS. The transplanted organoids labeled by PKH26 are presented red. Scale bar, 200 μm. **f** A representative confocal *Z*-stack image of the organoid-transplanted PCLS. Transplanted organoids labeled by PKH26 are presented in red. Blue, DAPI; red, PKH26. Scale bar, 100 μm. **g** Representative H&E images of the vertical section of control (top row) and organoid-transplanted (bottom row) PCLS. Scale bar, 50 μm. **h** Representative images of IF staining of organoid-transplanted PCLS (vertical section). Blue, DAPI; green, MSLN. Scale bar, 50 μm. **i** A representative confocal *Z*-stack image of the organoid-transplanted PCLS. The transplanted organoids labeled by PKH26 are presented red. Blue, DAPI; green, MSLN; red, PKH26. Scale bar, 100 μm. The data are presented as the means ± s.d. One-way ANOVA was used to determine significant differences among multiple groups. ***P < 0.001 versus the indicated group.

After successfully establishing pleura organoids, we investigated whether repairing damage to the visceral pleura could be achieved through organoid transplantation using both in vitro and in vivo experiments. Initially, we conducted in vitro wound healing assays to determine whether pleura organoids influence the migration and repair processes of mesothelial cells, the predominant cell type in the visceral pleura, following injury (Fig. 4a). The results indicated that transplanting pleura organoids into the upper layer of a Transwell system (2 × 10^4^ cells in group Graft1 and 5 × 10^4^ cells in group Graft2) enhanced mesothelial cell migration and increased the wound healing rate compared with the control group. These findings suggest that organoid transplantation has the potential to facilitate pleural repair (Fig. 4b, c). Subsequently, we established a PCLS model using immunodeficient NSG mice, which preserved the intact lung tissue structure (including the visceral pleura) and cellular diversity^32^. This model enabled us to evaluate the effect of organoid transplantation on pleural injury repair under conditions that closely mimic the native in vivo environment. After successfully culturing the PCLS, we used a surgical blade to create notches in the pleural region on the PCLS surface to simulate pleural rupture. We then applied PKH26 fluorescent dye-labeled human pleura organoid cells were added dropwise onto the injury site to simulate organoid transplantation (Fig. 4d). Using microscopy and confocal *Z*-stack imaging to detect PKH26 red fluorescence, we observed that the transplanted organoids successfully integrated into the damaged lung tissue (Fig. 4e, f). Furthermore, we selected the area with organoid cell integration and analyzed longitudinal PCLS sections via H&E staining. This analysis revealed that the transplanted organoid cells not only integrated into the damaged pleural area but also formed a continuous, intact and dense pleura-like structure on lung surface, consisting of multiple cell layers (Fig. 4g). In addition, immunofluorescence staining and confocal *Z*-stack imaging for MSLN demonstrated that the pleura-like structures formed after organoid transplantation were primarily composed of mesothelial cells (Fig. 4h, i).

### Organoid transplantation alleviates pleura injury in vivo

**Fig. 5:**
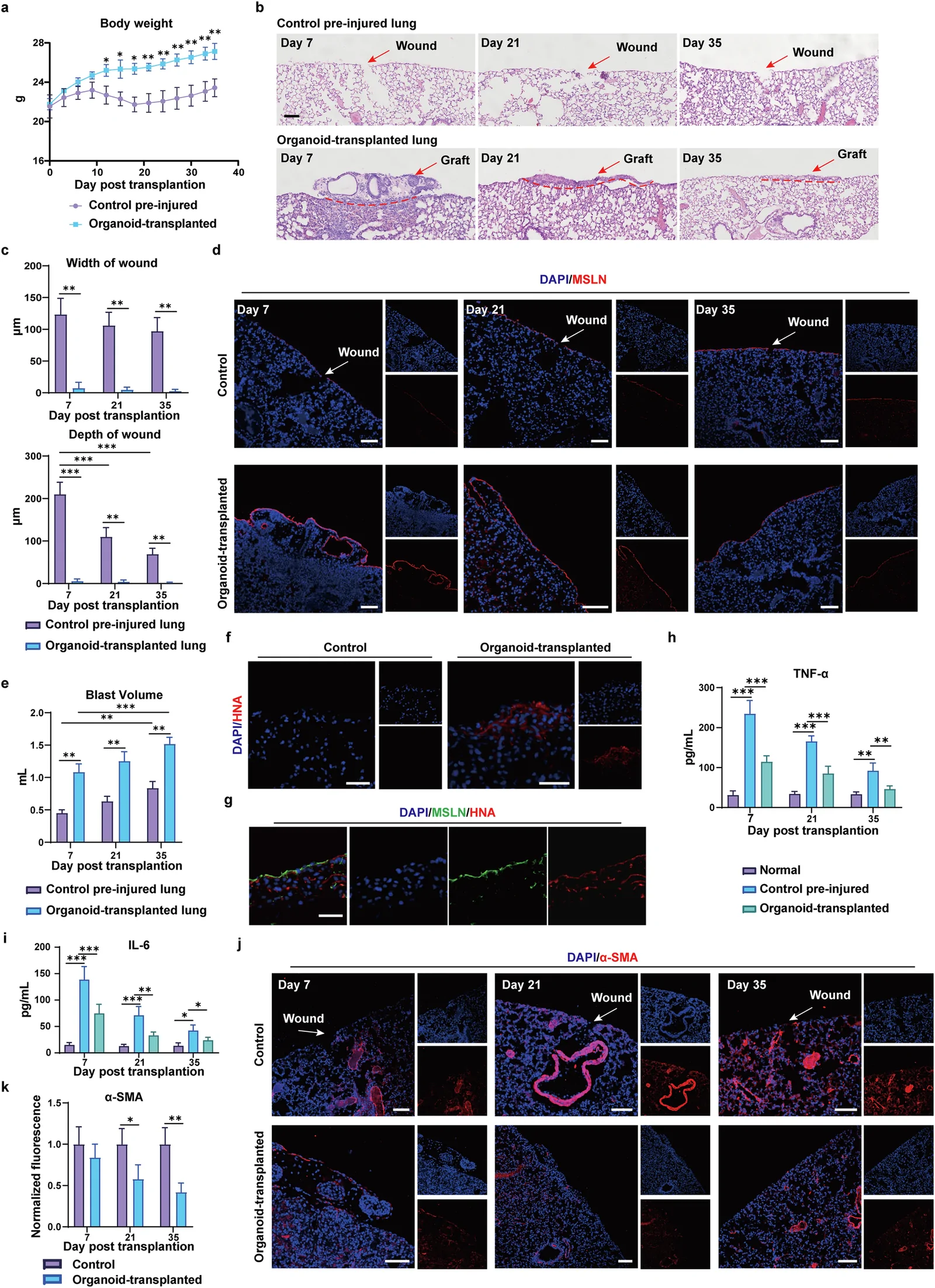
Organoids transplantation alleviates pleura injury in vivo. **a** The body weights of control pre-injured mice and organoid-transplanted mice from day 0 to day 35 post transplantation. *n* = 3 per group. **b** Representative H&E images of control pre-injured lung and organoid-transplanted lung at day 7, day 21 and day 35. Scale bar, 50 μm. **c** The quantification of the width and depth of wound in pre-injured lung and organoid-transplanted lung as described in **b**. *n* = 3 per group. **d** Representative images of IF staining of pre-injured organoid-transplanted lung at day 7, day 21 and day 35. Blue, DAPI; red, MSLN. Scale bar, 100 μm. **e** The quantification of the blast volumes of pre-injured lung and organoid-transplanted lung at day 7, day 21 and day 35. *n* = 3 per group. **f** Representative images of IF staining of pre-injured organoid-transplanted lung. Blue, DAPI; red, HNA. Scale bar, 50 μm. **g** Representative images of IF staining of pre-injured organoid-transplanted lung. Blue, DAPI; green, MSLN; red, HNA. Scale bar, 50 μm. **h**, **i** The comparison of TNF-α (**h**) and IL-6 (**i**) contents in the alveolar lavage fluid of healthy mice, pre-injured mice and organoid-transplanted mice at day 7, day 21 and day 35. *n* = 3 per group. **j** Representative images of IF staining of pre-injured organoid-transplanted lung at day 7, day 21 and day 35. Blue, DAPI; red, α-SMA. Scale bar, 100 μm. **k** The quantification of the normalized fluorescence intensity of the markers as described in **j**. *n* = 3 per group. The data are presented as the means ± s.d. Two-tailed unpaired *t*-tests were performed to assess the statistical significance of differences between two groups, whereas one-way ANOVA was used to determine significant differences among multiple groups. *P < 0.05, **P < 0.01 and ***P < 0.001 versus the indicated group.

After initially demonstrating the reparative effects of organoid transplantation on the visceral pleura through in vitro experiments, we proceeded to perform transplantation in a mouse model to further validate that organoid transplantation promotes the reparative process following pleural injury in vivo. We established a model of visceral pleura rupture in NSG immunodeficient mice via physico-mechanical disruption and subsequently transplanted cultured human pleura organoids into the pleural cavity. By contrast, control pre-injured mice received an equal volume of the same vector (50 μl pleura organoid medium containing 50% Matrigel and 10 µM Y-27632) without organoid cells. Our experimental design included three distinct time points—early (day 7), mid-term (day 21) and late-stage (day 35) post transplantation, enabling a detailed and continuous evaluation of pleural repair in both control and experimental groups. Supporting the overall therapeutic efficacy, mice in the experimental group exhibited a steady increase in body weight throughout the 35-day observation period, in contrast to a transient decline followed by a slow recovery observed in the model group (Fig. 5a). Histological analysis by H&E staining provided initial insights into morphological recovery. In the model group, although some wound healing from the lung parenchyma was observed by day 21 and day 35, significant surface wounds (in terms of width) persisted (Fig. 5b, c). Conversely, the experimental group demonstrated clear integration of the transplanted organoids into the lung surface as early as day 7, completely filling the wound area. At this early stage, the organoids largely retained their in vitro morphology. However, a remarkable transformation occurred by day 21, where the transplanted organoids matured into a dense, multilayered structure. Further maturation was evident by day 35, with this structure evolving into a looser 2–3-layered organization, structurally approaching the single-layer architecture characteristic of the native pleura (Fig. 5b, c).

Crucially, immunofluorescence staining for the mesothelial cell marker MSLN showed that transplanted organoids not only effectively integrated into the injured area but also rapidly formed a continuous mesothelial layer on the lung surface by day 7. This newly formed mesothelial layer remained intact and progressively integrated with the native mesothelial layer throughout the observation period, demonstrating remarkable stability up to day 35 (Fig. 5d). Conversely, the visceral pleura in the control group at day 35 still displayed unhealed wounds and lacked a continuous mesothelial lining. To functionally validate the observed wound healing and mesothelial remodeling, we conducted a quantitative assessment of pleural barrier integrity. We collected intact lungs with attached tracheas from mice post transplant, injected PBS into the trachea and measured the blast volume—the critical volume threshold causing PBS leakage—as an objective indicator of lung sealing. The experimental group consistently showed significantly higher blast volumes compared with the control group at all three time points (day 7, 21 and 35), demonstrating a robust and progressive improvement in pleural barrier function (Fig. 5e). Moreover, we detected immunofluorescence co-localization signals for HNA and MSLN on the surface of lung tissues from the transplantation group, indicating that the newly formed mesothelial layer was derived from transplanted organoids rather than mice’s own tissue regeneration (Fig. 5f, g).

To assess whether organoid transplantation could alleviate the inflammatory response caused by injury, we measured the levels of proinflammatory cytokines TNF-α and IL-6 in the alveolar lavage fluid of the mice. TNF-α and IL-6 levels in the model group showed a gradual decrease from early to late stages, they remained significantly elevated compared with normal controls even at day 35. Crucially, organoid transplantation significantly attenuated this acute inflammatory response from early stages, with TNF-α and IL-6 levels approaching those of the normal group by the late phase (Fig. 5h, i). Furthermore, we assessed the expression of α-SMA, a key marker for fibrosis, in the lung tissue. Our data reveal that organoid transplantation significantly attenuated α-SMA expression in the lung tissue from the middle to late stages (Fig. 5j, k), indicating a reduction in fibrotic progression. To verify the safety of pleura organoid transplantation, we performed histopathological analyses on the vital organs heart, liver, spleen and kidney of recipient mice. The results showed no obvious lesions or abnormalities in these organs and no obvious differences compared with control mice, confirming the safety of pleura organoid transplantation (Supplementary Fig. 1). These findings demonstrate the successful engraftment of transplanted pleural organoids into the injured lung surface, their progressive maturation into a continuous, single-layered mesothelial structure resembling the native pleura and the consequent restoration of pleural barrier integrity and attenuation of injury-induced inflammation and fibrosis.

### CD133^+^ cells are a stem cell-like population within pleura organoids

**Fig. 6:**
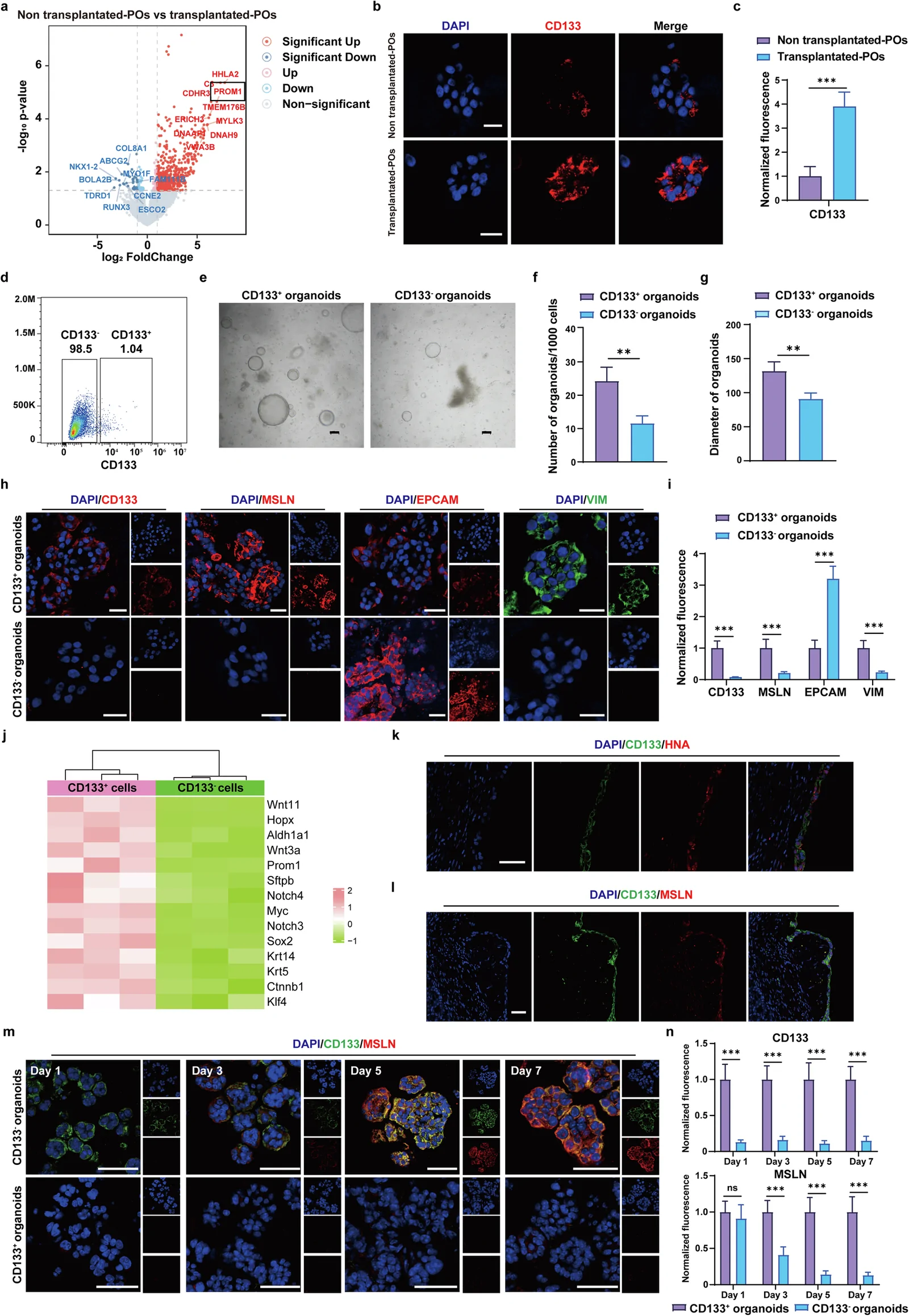
CD133^+^ cells are a stem cell-like population in pleura organoids. **a** A volcano plot showing the differential gene between nontransplanted pleura organoids (POs) and transplanted POs. *n* = 3 per group. **b** Representative images of IF staining of nontransplanted POs and transplanted POs. Blue, DAPI; red, CD133. Scale bar, 20 μm. **c** The quantification of the normalized fluorescence intensity of CD133 as described in **b**. *n* = 3 per group. **d** A representative flow cytometry plot showing the percentages of sorted CD133^+^ cells and CD133^−^ cells from pleura organoids. **e** Representative brightfield microscopy images of CD133^+^ cell-derived and CD133^−^ cell-derived organoids. Scale bars, 100 μm. **f**, **g** The quantification of the numbers (**f**) and diameters (**g**) of CD133^+^ cell-derived and CD133^−^ cell-derived organoids. *n* = 3 per group. **h** Representative images of IF staining of CD133^+^ cell-derived and CD133^−^ cell-derived organoids. Blue, DAPI; red, CD133; red, MSLN; red, EPCAM; green, VIM. Scale bar, 20 μm. **i** The quantification of the normalized fluorescence intensity of the markers as described in **h**. *n* = 3 per group. **j**, A heat map of DEGs associated with stemness between CD133⁺ cells and CD133⁻ cells. **k**, **l** Representative images of IF staining of organoid-transplanted kidney: blue, DAPI; green, CD133; red, HNA (**k**); blue, DAPI; green, CD133; red, MSLN (**l**). Scale bar, 50 μm. **m** Representative images of IF staining of CD133^+^ cell-derived and CD133^−^ cell-derived organoids at day 1, day 3, day 5 and day 7. Blue, DAPI; green, CD133; red, MSLN. Scale bar, 50 μm. **n** The quantification of the normalized fluorescence intensity of the markers as described in **m**. *n* = 3 per group. The data are presented as the means ± s.d. Two-tailed unpaired *t*-tests were performed to assess the statistical significance of differences between two groups. **P < 0.01 and ***P < 0.001 versus the indicated group.

After demonstrating through in vitro and in vivo experiments that organoid transplantation can effectively repair pleural damage, we investigated the potential mechanisms involved in pleural repair following this transplantation. The pleura organoids we constructed contained multiple cellular components, with different cell types exhibiting distinct biological characteristics, such as proliferation and differentiation potential. This variability suggests that these cells may contribute differently to the repair process following pleural injury. Therefore, our objective was to identify the subpopulation of cells primarily involved in the injury repair process. To achieve this, we collected pleura organoids integrated into lung tissue and involved in injury repair after transplantation (transplanted pleura organoids; this subset of organoids has been functionally enriched in vivo and theoretically contains a higher proportion of functional cell subpopulations responsible for injury repair) and compared them with those that did not undergo transplantation (nontransplanted pleura organoids). We analyzed the gene expression profiles of the functional cell subpopulations using transcriptome sequencing and explored potential identifying markers. Differential gene analysis revealed that CD133 (also known as PROM1) was highly expressed in transplanted pleura organoids (Fig. 6a). Given that the *CD133* gene has been reported to be expressed in various stem and progenitor cells in previous studies and is involved in maintaining the ‘stemness’ of stem/progenitor cell^22^, we hypothesized that CD133^+^ cells may represent a functional subpopulation involved in the repair of pleural injuries following organoid transplantation. Immunofluorescence staining revealed that the expression of CD133 was significantly elevated in the transplanted organoids compared with that in the nontransplanted organoids (Fig. 6b, c).

To determine whether the CD133^+^ cells exhibit stem cell characteristics, we isolated CD133^+^ and CD133^−^ subpopulations from pleura organoids using flow cytometry and assessed their organoid-forming capabilities (Fig. 6d). We generated pleura organoids from both CD133^+^ and CD133^−^ cells as initial sources and found that CD133^+^ cells demonstrated a significantly greater capacity to form organoids and proliferated more efficiently (Fig. 6e–g). Moreover, organoids derived from CD133^+^ contained multiple cell types, including MSLN^+^ mesothelial cells, EPCAM^+^ epithelial cells and VIM^+^ mesenchymal cells or fibroblasts, indicating the multilineage differentiation potential of CD133^+^ cells (Fig. 6h, i). By contrast, CD133^−^ cells were restricted to differentiating into epithelial cells. To better understand the functional aspects of the CD133^+^ subpopulation in pleural organoids, we proceeded to isolate CD133^+^ and CD133^−^ cells from these organoids and performed transcriptome sequencing (RNA-seq) analysis. Our analysis identified 1,457 differentially expressed genes (DEGs) between the CD133^+^ and CD133^−^ populations, and we observed the upregulation of several genes associated with stemness maintenance in the CD133^+^ cell population (Fig. 6j and Supplementary Fig. 2a,b). Gene Ontology (GO) analysis of DEGs revealed significant enrichment in molecular functions and biological processes strongly associated with cell division, including mitotic sister chromatid segregation, chromosome segregation and nuclear chromosome segregation (Supplementary Fig. 2c). This finding is highly consistent with our observed significant differences in organoid-forming capacity and proliferative potential between CD113^+^ and CD133^−^ cells. Furthermore, Kyoto Encyclopedia of Genes and Genomes (KEGG) pathway analysis showed significant enrichment in metabolism-related pathways, such as drug metabolism—cytochrome P450, arachidonic acid metabolism and glutathione metabolism, suggesting distinct metabolic characteristics for CD133^+^ cells (Supplementary Fig. 2d).

In addition, we observed CD133 expression in pleura-like structures formed after transplantation of CD133^+^ organoids beneath the kidney capsule (Fig. 6k). Notably, CD133^+^ cells co-expressed the mesothelial cell marker MSLN, suggesting their potential to differentiate into mesothelial cells in vivo (Fig. 6l). To determine whether the CD133⁺ cells directly give rise to mesothelial cells, we performed a simplified in vitro lineage tracing experiment. CD133⁺ and CD133⁻ cell-derived organoids were collected at day 1, day 3, day 5 and day 7 to dynamically track the emergence of mesothelial cells by detecting MSLN expression. The results demonstrated that CD133⁺ organoids exhibited minimal MSLN expression on day 1, with detectable levels appearing by day 3 and widespread MSLN expression observed throughout the organoids by day 5 and day 7. By contrast, under identical induction conditions, CD133⁻ cells almost completely failed to differentiate into MSLN⁺ mesothelial cells (Fig. 6m, n). These in vitro findings provide compelling evidence that CD133⁺ cells possess the intrinsic capacity to differentiate into MSLN⁺ mesothelial cells, rather than merely co-expressing mesothelial markers. This robust in vitro differentiation capability leads us to hypothesize that CD133⁺ cells may also possess the potential to generate mesothelial cells in vivo.

These results indicate that CD133^+^ cells are enriched in organoids involved in pleural repair post transplantation and possess several stem cell properties, as evidenced by their enhanced proliferation, multilineage differentiation potential and expression of genes associated with stemness maintenance. Therefore, CD133^+^ cells probably represent a functional population with some stem cell characteristics within pleura organoids.

### scRNA-seq analysis identifies a distinct CD133^+^ population in human lung

**Fig. 7:**
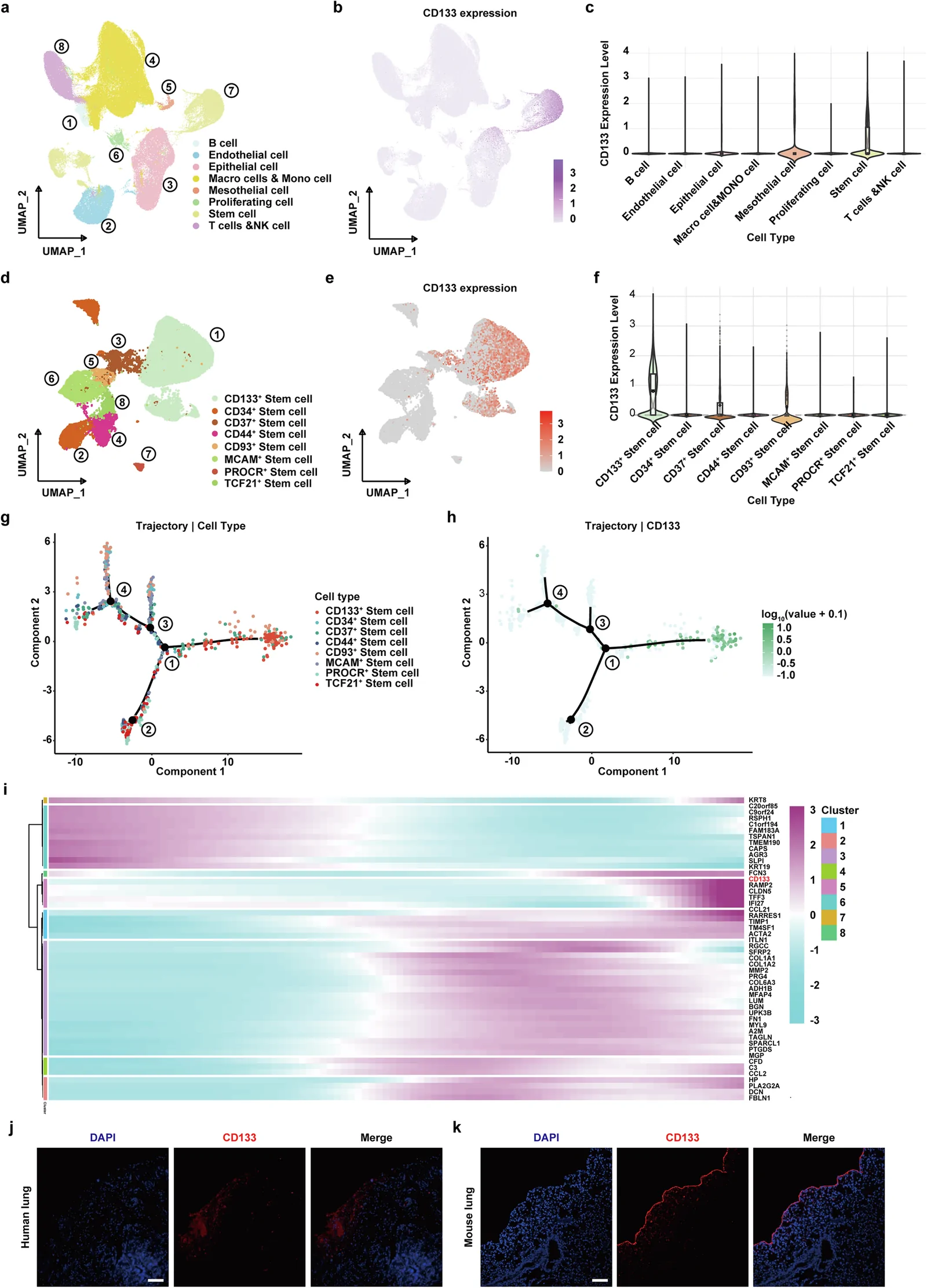
scRNA-seq analysis identifies a distinct CD133^+^ population in human lung. **a** A UMAP plot showing the annotated eight major cell types. **b**, **c** The UMAP (**b**) and violin (**c**) plots displaying the expression levels of *CD133* gene across annotated clusters. **d** UMAP plots showing the annotated eight stem cell clusters. **e**, **f** The UMAP (**e**) and violin (**f**) plots displaying the expression levels of *CD133* gene across annotated eight stem cell clusters. **g** The pseudotime differentiation trajectory of stem cell clusters. **h** The pseudotime differentiation trajectory of *CD133* gene across stem cell clusters. **i** A heat map showing the differentiation trajectory of gene expression of stem cell clusters. **j**, **k** Representative images of IF staining of human (**j**) and mouse (**k**) lung. Blue, DAPI; red, CD133. Scale bar, 100 μm.

To determine if a distinct CD133^+^ cell population exists within pleural tissue, we leveraged publicly available scRNA-seq data of human lung tissue from GEO database. After performing quality control analysis (Supplementary Fig. 3), we identified 33 distinct cell clusters (0–32) on the basis of their transcriptional profiles by using *t*-SNE dimensionality reduction (Supplementary Fig. 4a). On the basis of the expression of classic marker genes, these clusters were subsequently annotated into eight major cell types, including B cells, endothelial cells, epithelial cells, macrophages and monocytes, mesothelial cells, proliferative cells, stem cells and T cells and NK cells. Each cell type occupied distinct regions in the *t*-SNE plot (Fig. 7a). The cell type identity and specificity were further validated by marker gene expression: representative markers such as the epithelial marker *EPCAM*, endothelial marker *VWF* and stem cell marker *SOX2* showed clear cell type-restricted expression patterns (Supplementary Fig. 4b). Consistent with our previous conclusion that CD133^+^ cells harbor several stem cell features, both *t*-SNE visualization and violin plots confirmed that *CD133* was significantly enriched in identified stem cell cluster (Fig. 7b, c).

To further explore the heterogeneity within this stem cell population, we performed subclustering on the basis of their gene expression profiles (Supplementary Fig. 4c), which resolved into eight distinct stem cell subpopulations (Fig. 7d and Supplementary Fig. 4d). Importantly, *CD133* expression was highly enriched in a specific subpopulation defined as CD133^+^ stem cell cluster (Fig. 7e, f). GO and KEGG pathway enrichment analysis revealed that this CD133^+^ stem cell subcluster harbors a unique metabolic transcriptomic profile, with significant enrichment of biological processes and pathways including fatty acid metabolic process, amino acid metabolic process, carbon metabolism and propanoate metabolism (Supplementary Fig. 4e,f).

To delineate potential dynamic transitions between stem cell states, we constructed a pseudotime differentiation trajectory. This analysis showed that the stem cell differentiation trajectory branched into four nodes, with the CD133^+^ stem cell subpopulation positioned at a terminal point of this trajectory (Fig. 7g). Consistent with this, *CD133* expression levels progressively increased from earlier stem cell subpopulations toward the CD133^+^ stem cell cluster along the pseudotime trajectory (Fig. 7h, i). Furthermore, we performed subclustering of the mesothelial cell population (Supplementary Fig. 5a,b) and identified a CD133^+^ mesothelial cell subpopulation where *CD133* was relatively enriched (Supplementary Fig. 5c,d). This observation suggests a potential developmental link or differentiation trajectory from CD133^+^ stem cells toward certain mesothelial cell fates, a hypothesis that warrants further investigation. To provide direct visual evidence, we performed immunofluorescence staining for CD133^+^ cells in both human and mouse lung tissues under homeostatic conditions. Our results clearly demonstrate the presence of CD133^+^ cells, predominantly localized along and adjacent to the pleura (Fig. 7j, k).

Collectively, these results demonstrate that CD133^+^ cells with stem cell-like gene expression profiles and unique metabolic characteristics do exist in the pleural region of lung tissues under homeostatic conditions. On the basis of our current data, CD133^+^ cells primarily resemble a resident stem cell-like population under physiological homeostasis, rather than a transiently amplifying population that emerges during injury repair.

### CD133^+^ cells are the key functional population promoting pleura injury repair after organoids transplantation

**Fig. 8:**
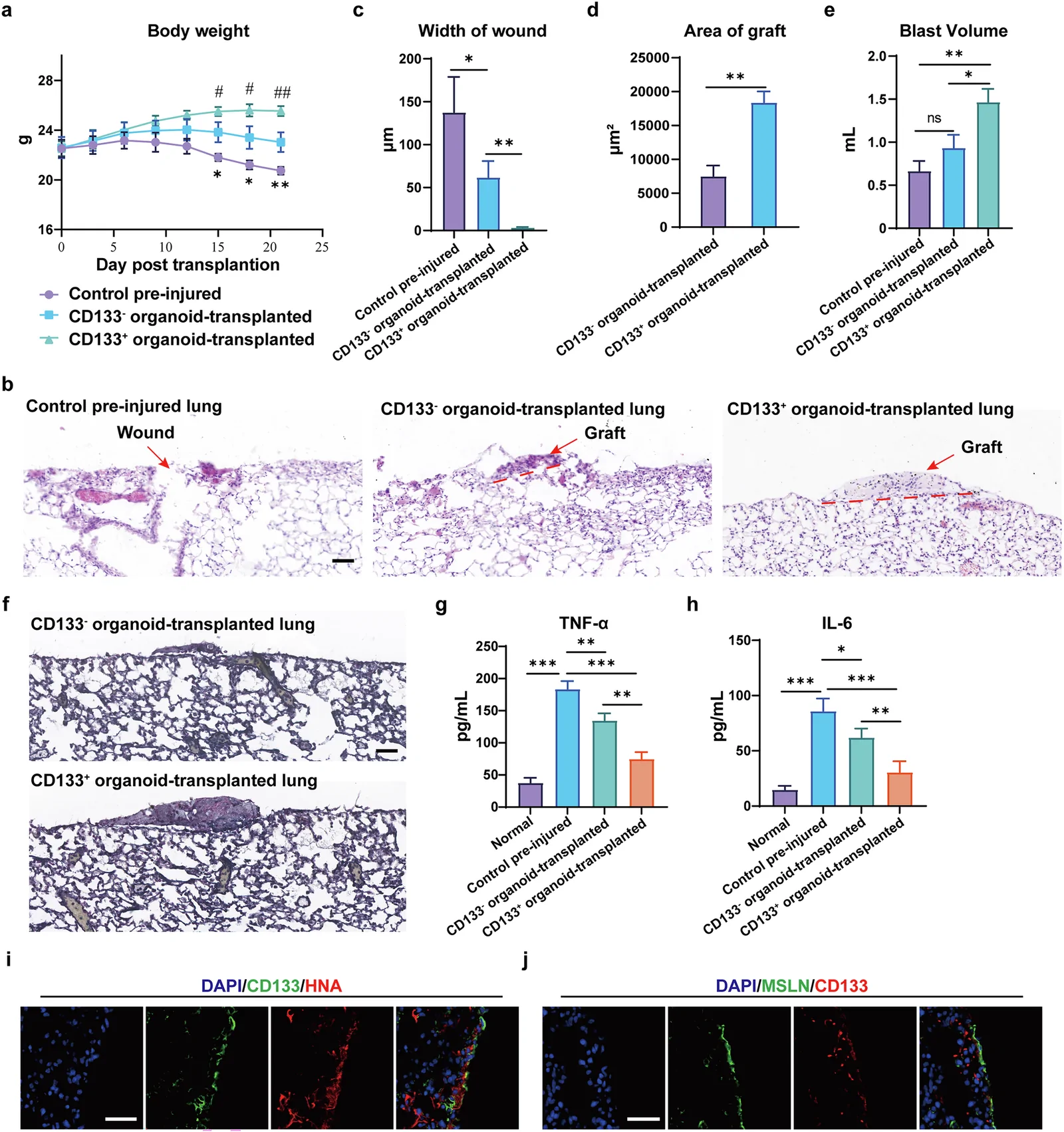
CD133^+^ cells are the key functional population promoting pleura injury repair after organoids transplantation. **a** The body weights of control pre-injured mice, CD133^−^ organoid-transplanted mice and CD133^+^ organoid-transplanted mice from day 0 to day 21 post transplantation. *n* = 3 per group. **b** Representative H&E images of control pre-injured lung, CD133^−^ organoid-transplanted lung and CD133^+^ organoid-transplanted lung. Scale bar, 50 μm. **c** The quantification of the wound width in control pre-injured lung, CD133^−^ organoid-transplanted lung and CD133^+^ organoid-transplanted lung. *n* = 3 per group. **d** The quantification of the areas of grafts in CD133^−^ organoid-transplanted lung and CD133^+^ organoid-transplanted lung. *n* = 3 per group. **e** The quantification of the blast volumes of control pre-injured lung, CD133^−^ organoid-transplanted lung and CD133^+^ organoid-transplanted lung. *n* = 3 per group. **f** Representative images of EVG-Verhöeff staining of CD133^−^ organoid-transplanted lung and CD133^+^ organoid-transplanted lung. Scale bar, 50 μm. **g**, **h** A comparison of TNF-α (**g**) and IL-6 (**h**) contents in the alveolar lavage fluid of healthy mice, pre-injured mice, CD133^−^ organoid-transplanted mice and CD133^+^ organoid-transplanted mice. *n* = 3 per group. **i** Representative images of IF staining of CD133^+^ organoid-transplanted lung. Blue, DAPI; green, CD133; red, HNA. Scale bar, 50 μm. **j** Representative images of IF staining of CD133^+^ organoid-transplanted lung. Blue, DAPI; green, MSLN; red, CD133. Scale bar, 50 μm. The data are presented as the means ± s.d. Two-tailed unpaired *t*-tests were performed to assess the statistical significance of differences between two groups, whereas one-way ANOVA was used to determine significant differences among multiple groups. *P < 0.05, **P < 0.01 and ***P < 0.001 versus the indicated group.

To demonstrate the role of CD133^+^ cells in pleural injury repair, CD133^+^ and CD133^−^ cell populations from human pleura organoids were sorted and cultured for in vivo transplantation experiments. An equal number (5 × 10^5^ cells) of CD133^+^ or CD133^−^ organoids were transplanted into the pleural cavity of NSG immunodeficient mice with pleural rupture. Control pre-injured mice received an equal volume of the same vector without organoid cells. Within 21 days post transplantation, mice in the control pre-injured group exhibited gradual weight loss. By contrast, transplantation of CD133^−^ organoids alleviated the weight loss caused by pleural injury, whereas transplantation of CD133^+^ organoids nearly maintained body weight (Fig. 8a). Histological examination using H&E staining revealed marked damage to the visceral pleura of control pre-injured mice 21 days after the experiment began. In the CD133^−^ organoids-transplanted group, the organoids showed limited repair capability, as evidenced by reduced wound width but no significant improvement in pleura integrity (Fig. 8b–d). By contrast, transplantation of CD133^+^ organoids resulted in markedly enhanced repair. Larger grafts were observed on the surface of lungs transplanted with CD133^+^ organoids, forming a continuous and intact structure over pleural damage area, which nearly completely repairing the pleural wound. Moreover, the CD133^+^ organoid-transplanted lungs exhibited a significantly increased blast volume compared with those transplanted with CD133^−^ organoids, indicating improved sealing of the pleura (Fig. 8e). The distribution of collagen and elastic fibers within these multilayered structures was detected by EVG-Verhöeff staining, demonstrating extracellular matrix remodeling following CD133^+^ organoid transplantation (Fig. 8f). To assess whether organoid transplantation could alleviate the inflammatory response caused by injury, we measured TNF-α and IL-6 levels in the alveolar lavage fluid. Both cytokines were significantly elevated in control pre-injured mice compared with normal mice. The transplantation of CD133^+^ organoids significantly attenuated this inflammatory response, whereas whereas CD133^−^ organoids were notably less effective (Fig. 8g, h).

Furthermore, in lungs transplanted with CD133^+^ organoids, CD133 expression was detected, showing a tendency to localize near the visceral pleura (Fig. 8i). Notably, CD133^+^ cells co-expressed the mesothelial cell marker MSLN in the surface layer (Fig. 8j), suggesting their potential contribution to mesothelial cell formation and integration within the regenerated tissue. These in vivo observations indicate that CD133^+^ cells, as a key functional population, contribute significantly to the structural repair of the visceral pleura, mesothelial regeneration and acute inflammation resolution during the repair process following pleura organoid transplantation, but whether their contribution involves direct differentiation into mesothelial cells in vivo still requires further definitive confirmation.

## Discussion

PAL following thoracic surgery remains a critical clinical challenge, necessitating novel strategies that move beyond palliative interventions to promote true tissue regeneration. This study addresses this unmet clinical need by establishing pleura organoids as a novel regenerative therapy. We demonstrate their unique capacity to dynamically regenerate pleural structures, restore tissue integrity and re-establish pleural barrier function in preclinical models. Our findings redefine regenerative strategies for PAL by integrating robust cellular regeneration with progressive tissue architectural restoration, while identifying CD133^+^ cells as a critical subpopulation contributing to mesothelial repair, a previously unrecognized mechanism.

A central achievement of this work is the development of pleura-specific organoids that accurately replicate the structural and functional characteristics of native pleura. Unlike lung organoids, which primarily model alveolar or airway components^33,34^, our pleura organoids consistently express mesothelial markers (MSLN and CR) and stromal markers (VIM), exhibiting robust proliferative activity throughout extended culture. Their distinct morphology, characterized by dense outer layers and cystic cores, reflects an adaptation to the biomechanical demands of the pleural microenvironment, where elasticity and tensile strength are critical. Remarkably, xenotransplantation beneath the murine kidney capsule produced stratified tissues that mimic human visceral pleura, featuring organized mesothelial monolayers, collagen networks and elastic fibers. This structural fidelity, validated by EVG-Verhöeff staining and human-specific HNA tracing, highlights the capacity of organoids to reconstruct the multilayered architecture of the pleura, a feat that synthetic patches or chemical adhesives cannot fully achieve^35–37^.

The identification of CD133^+^ cells as a regenerative subpopulation offers significant insights into pleural repair mechanisms. The transcriptomic analysis of human lung single-cell datasets further supports the enrichment of *CD133* gene in stem cell and mesothelial populations, aligning with our findings. Our functional analyses demonstrate that CD133^+^ cells exhibit a significantly enhanced capacity for organoid formation and robust proliferation in vitro. Crucially, through in vitro lineage tracing experiments, we observed their intrinsic capacity to differentiate into MSLN^+^ mesothelial cells over time, a capability largely absent in CD133^−^ cells. Transcriptomic profiling further revealed an enrichment of genes associated with stemness maintenance, cell division and distinct metabolic pathways within the CD133^+^ population, consistent with their potent proliferative and multilineage differentiation potential in vitro. Although co-localization of CD133 and MSLN in transplanted organoids suggests their contribution to re-mesothelialization in vivo, we acknowledge that definitive in vivo lineage tracing or specific loss-of-function validation is warranted to fully confirm their direct differentiation and self-renewal capacity. Nevertheless, these compelling in vitro characteristics, combined with their strong association with repair in vivo, position CD133^+^ cells as a critical population within pleura organoids, actively contributing to and facilitating the observed tissue regeneration.

Our integrated models provide robust evidence supporting the therapeutic potential of pleura organoids. In vitro, the PCLS-based wound healing assay demonstrated that organoids enhance mesothelial cell migration and integration into injury sites. In vivo, transplantation into a murine pleural rupture model resulted in dynamic structural restoration, characterized by progressive maturation of the graft into a continuous, native-like mesothelial layer and sustained recovery of pleural barrier function over 35 days. This was quantitatively demonstrated by significantly increased blast volumes and robust histological evidence of continuous mesothelial layer reformation and integration with the host tissue. Importantly, this approach not only facilitated structural repair but also significantly attenuated acute inflammation, as evidenced by reduced levels of TNF-α and IL-6 in alveolar lavage fluid. This indicates a biologically precise alternative to current palliative strategies that often trigger inflammatory cascades through synthetic materials or chronic adhesions^38,39^, moving beyond mere mechanical closure to organized tissue repair.

This work represents the first demonstration of pleura organoids as a regenerative tool for visceral pleural injury, addressing a critical gap in thoracic regenerative medicine. By harnessing pleura organoids, we achieve not only continuous mesothelial regeneration but also facilitate the progressive restoration of functional tissue architecture, offering a multifaceted repair mechanism largely unattainable with existing therapies. Clinically, this strategy aligns with the paradigm of personalized medicine: patient-specific pleural biopsies could potentially generate autologous organoids, mitigating immune rejection risks. Furthermore, the demonstrated scalability of organoid production (exceeding 20 passages) highlight the translational feasibility of this approach. Although our organoids primarily model the mesothelial and stromal components of the pleura, fully recapitulating the intricate cellular architecture and interactions with distal lung cells, such as alveolar epithelial cells, remains a challenging but promising future direction requiring advanced bioengineering approaches.

Although challenges such as immune compatibility and large-scale bioengineering persist^40,41^, our study establishes a foundation for overcoming these obstacles. Future efforts could explore autologous induced pluripotent stem cell-derived organoids or biomaterial-enhanced delivery systems to optimize clinical applications. Nevertheless, the immediate significance of this work lies in its proof of concept: pleura organoid transplantation effectively restores pulmonary homeostasis through a progenitor-driven mechanism, providing a transformative solution for a complication that prolongs suffering and increases healthcare costs.

This study redefines visceral pleural repair by integrating regenerative biology with surgical innovation. By elucidating the role of CD133^+^ cells and establishing pleura organoids as a versatile therapeutic platform, we provide a road map for next-generation therapies that prioritize biological fidelity over purely mechanical intervention.

## Supplementary information

Supplementary Information
