# Supplementary material for: Pleura organoid transplantation facilitates visceral pleural repair through CD133+ progenitor cell-mediated regeneration
Source: Exp Mol Med. 2026 Sep 3;58(9):2990–3006. doi: 10.1038/s12276-026-01825-w (PMC13633260; doi:10.1038/s12276-026-01825-w)
Supplement: Supplementary file 1 [file 12276_2026_1825_MOESM1_ESM.pdf]

Supplementary Information

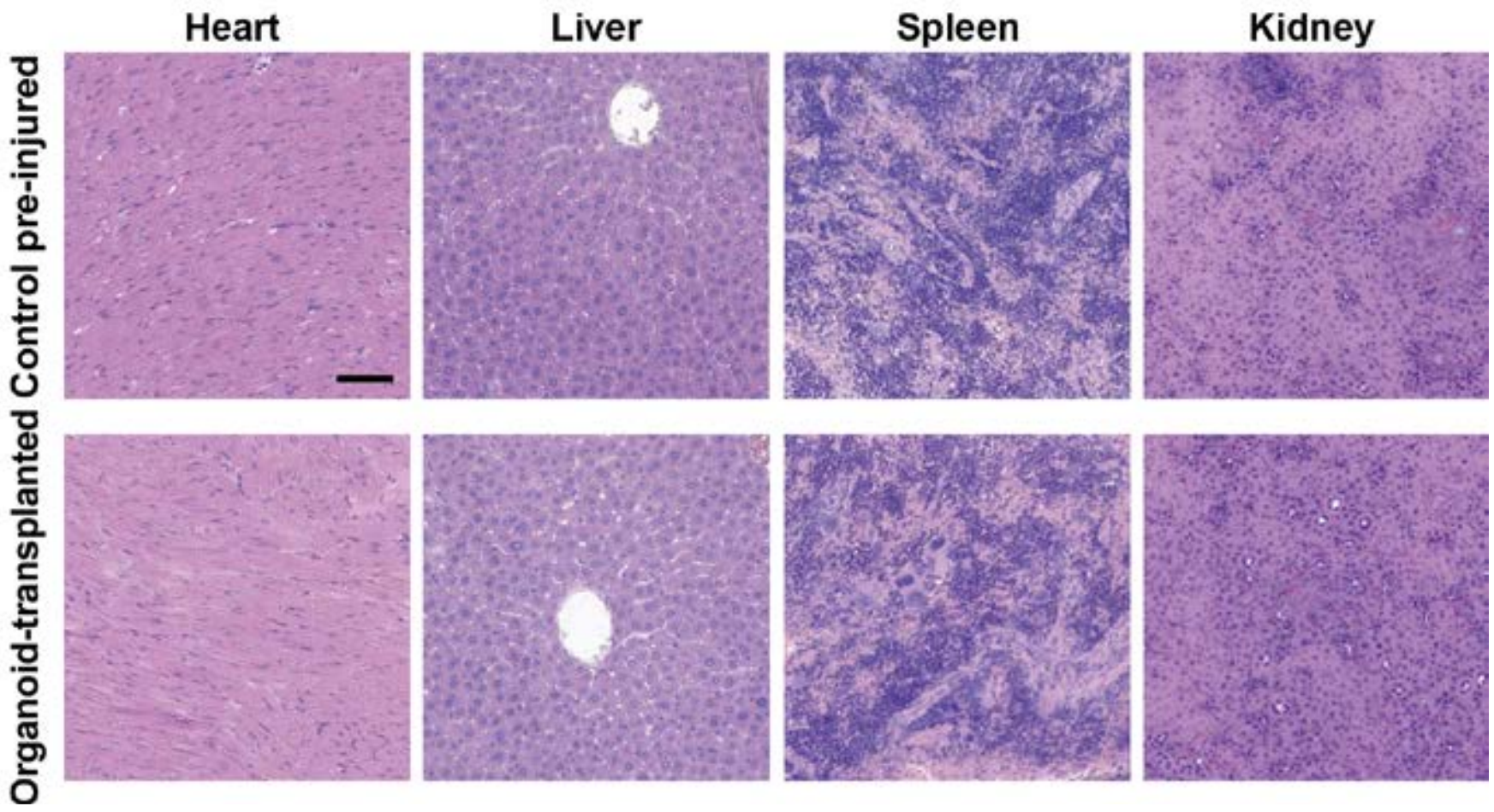

Supplementary Fig. 1 Safety assessment of pleura organoid transplantation. Representative H&E images of hearts, livers, spleens and kidneys of control pre-injured mice and organoid-transplanted mice. Scale bar, 50 µm.

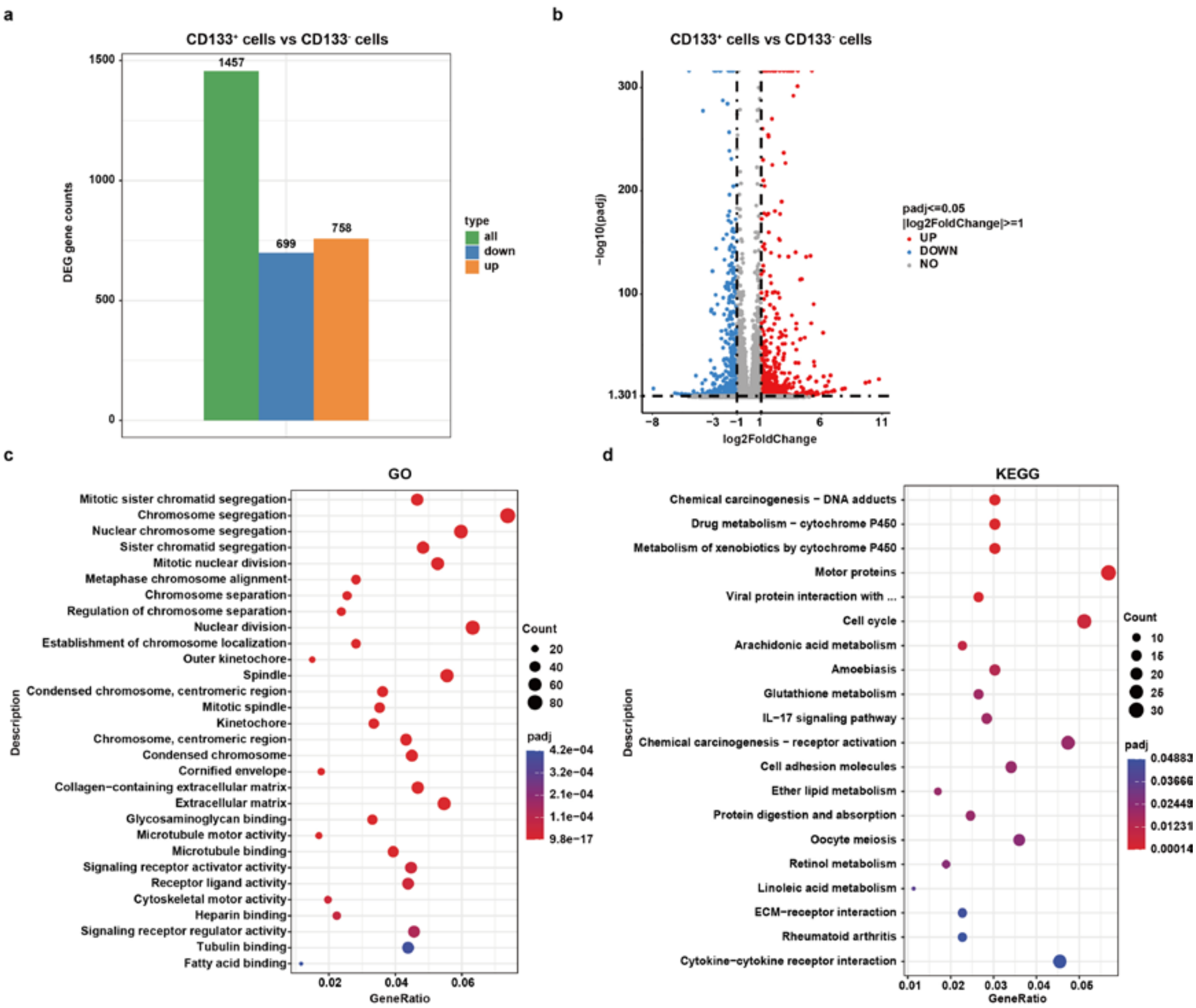

Supplementary Fig. 2 The RNA-seq analysis of CD133^{+} and CD133^{-} cells from pleura organoids. a, b Statistics plot (**a**) and volcano plot (**b**) showing the differentially expressed genes (DEGs) between CD113^{+} and CD133^{-} cells. n = 3 per group. **c** GO enrichment analysis of the DEGs between CD113^{+} and CD133^{-} cells. **d** KEGG enrichment analysis of the DEGs between CD113^{+} and CD133^{-} cells.

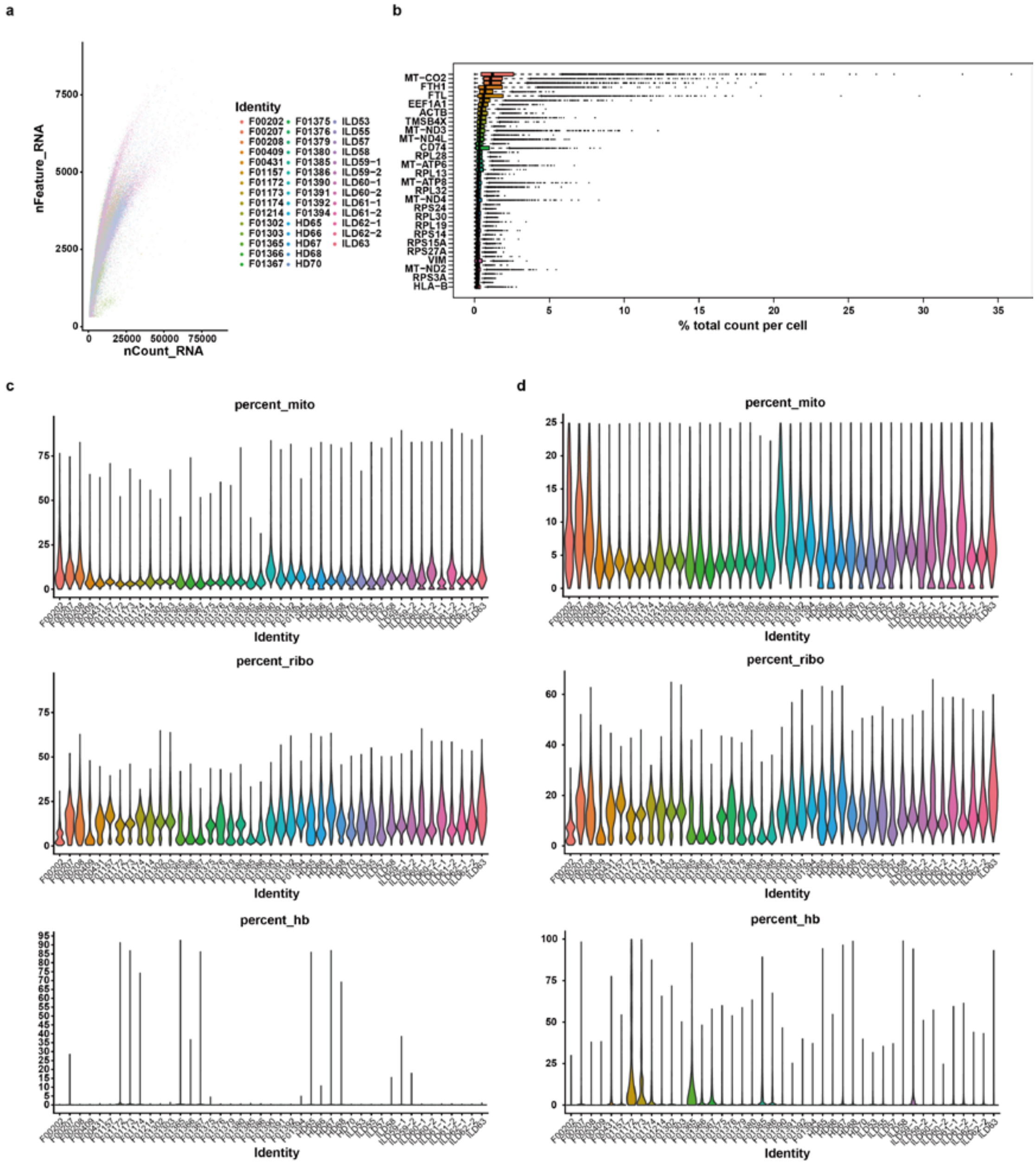

Supplementary Fig. 3 Quality control analysis of single-cell RNA sequencing (scRNA-seq) data. a Scatter plot showing the number of unique genes detected per cell (nFeature_RNA) versus the total number of unique molecular identifiers per cell (nCount_RNA), coloured by sample identity. **b** Box plot displaying the top genes contributing to the highest percentage of total counts per cell. **c, d** Violin plots depicting the percentage of mitochondrial gene reads (percent_mito, top), ribosomal gene reads (percent_ribo, middle), and hemoglobin gene reads (percent_hb, bottom) per cell for each individual sample (Identity) before (**c**) and after (**d**) filtering.

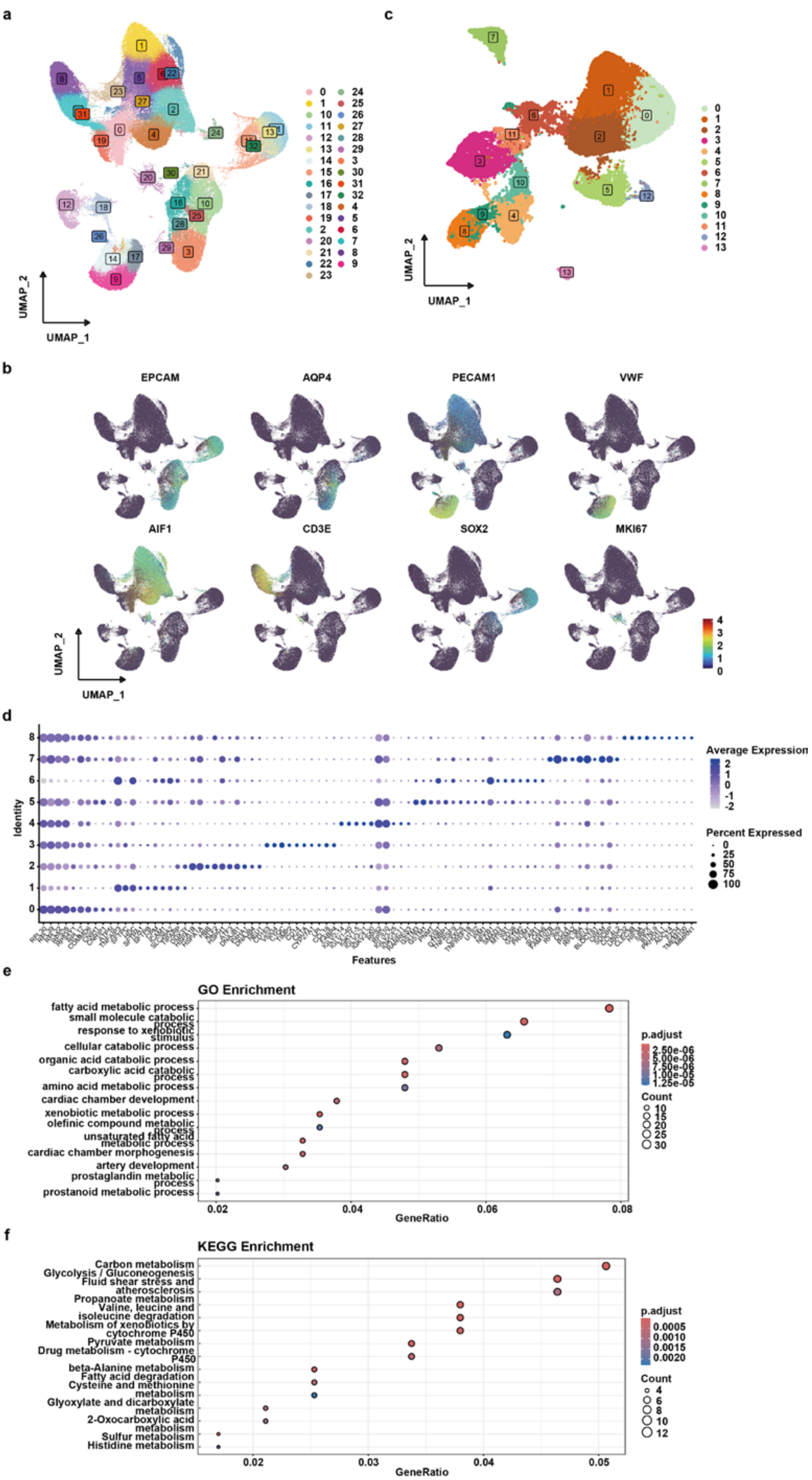
a
UMAP_2
UMAP_1
c
UMAP_2
UMAP_1
b
EPCAM
AQP4
PECAM1
VWF
AIF1
CD3E
SOX2
MKI67
UMAP_2
UMAP_1
d
Identity
Features
Average Expression
Percent Expressed
e
GO Enrichment
fatty acid metabolic process
small molecule catabolic process
response to xenobiotic stimulus
cellular catabolic process
organic acid catabolic process
carboxylic acid catabolic process
amino acid metabolic process
cardiac chamber development
xenobiotic metabolic process
olefinic compound metabolic process
unsaturated fatty acid metabolic process
cardiac chamber morphogenesis
artery development
prostaglandin metabolic process
prostanoid metabolic process
GeneRatio
p.adjust
Count
f
KEGG Enrichment
Carbon metabolism
Glycolysis / Gluconeogenesis
Fluid shear stress and atherosclerosis
Propanoate metabolism
Valine, leucine and isoleucine degradation
Metabolism of xenobiotics by cytochrome P450
Pyruvate metabolism
Drug metabolism - cytochrome P450
beta-Alanine metabolism
Fatty acid degradation
Cysteine and methionine metabolism
Glyoxylate and dicarboxylate metabolism
2-Oxocarboxylic acid metabolism
Sulfur metabolism
Histidine metabolism
GeneRatio
p.adjust
Count

Supplementary Fig. 4 scRNA-seq analysis of human lung tissue. a UMAP plot of scRNA-seq data from human lung tissue, coloured by cluster identity. **b** UMAP plots displaying the expression levels of key cell type-specific marker genes across all cells. **c** UMAP plot showing the refined clustering of stem cell cluster. **d** Dot plot displaying the average expression (colour gradient) and percentage of cells expressing (dot size) selected feature genes across the 9 stem cell identities. **e, f** GO (**e**) and KEGG (**f**) enrichment analysis of CD133$^+$ stem cell cluster-specific marker genes.

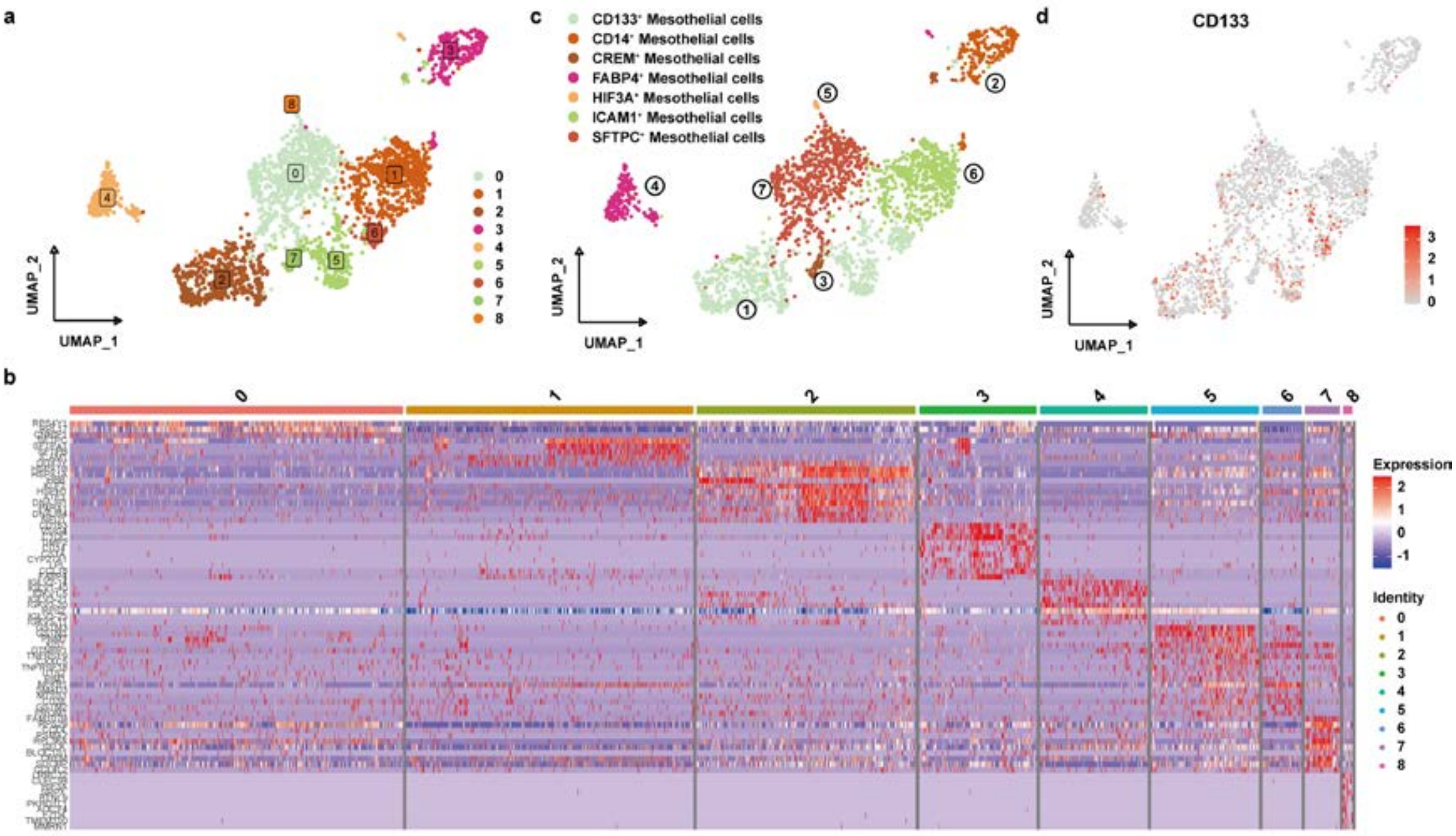

Supplementary Fig. 5 Sub-clustering and molecular characterization of human lung mesothelial cell heterogeneity. a UMAP plot showing the refined clustering of mesothelial cell cluster. **b** Heatmap illustrating the normalized expression levels of key marker genes (rows) across individual mesothelial cells (columns). **c** Annotation of mesothelial cell subtypes based on marker gene expression. **d** UMAP plot showing the normalized expression levels of the *CD133* gene across the entire mesothelial cell population.

Supplementary Table 1. Media formulation used in this study.

Components	Murine lung organoid	Murine pleura organoid	Human lung organoid	Human pleura organoid
Basal medium	advanced DMEM/F-12	Medium 199	advanced DMEM/F-12	Medium 199
GlutaMAX	2 mM	2 mM	2 mM	2 mM
N-acetylcysteine	1 mM	1 mM	1 mM	1 mM
Nicotinamide	-	-	1 mM	1 mM
B27 supplement	2%	2%	2%	2%
N2 supplement	2%	2%	2%	2%
RSPO1	100 ng/mL	100 ng/mL	100 ng/mL	100 ng/mL
EGF	50 ng/mL	10 ng/mL	50 ng/mL	10 ng/mL
FGF-10	100 ng/mL	20 ng/mL	100 ng/mL	20 ng/mL
FGF-7	25 ng/mL	-	50 ng/mL	-
Noggin	100 ng/mL	100 ng/mL	100 ng/mL	100 ng/mL
Wnt-3A conditioned medium	10%	10%	10%	10%
Y-27632	10 µM	10 µM	10 µM	10 µM
A83-01	-	2 µM	-	2 µM
Ascorbic acid	-	50 µg/mL	-	50 µg/mL
Prostaglandin E2	-	-	2 µM	2 µM
ITS	-	1%	-	1%
Dexamethasone	50 nM	200 nM	50 nM	200 nM
8-bromo-cAMP	100 nM	-	100 nM	-
Retinoic acid	-	50 nM	-	50 nM
IBMX	100 µM	-	100 µM	-
